# Slowed Gompertzian ageing in long-lived *C. elegans* results from expansion of decrepitude, not decelerated ageing

**DOI:** 10.1038/s41467-026-71780-7

**Published:** 2026-04-13

**Authors:** Bruce Zhang, David Gems

**Affiliations:** Institute of Healthy Ageing, and Research Department of Genetics, Evolution and Environment, University College London, London, UK

**Keywords:** Ageing, Population dynamics, Epidemiology

## Abstract

In populations of many animal species, including humans, mortality rates increase exponentially with advancing age. The scale and rate of increase can be set by two parameters, *α* and *β*, respectively, of the Gompertz equation. Interventions that extend lifespan can reduce either or both parameters. A long-standing supposition is that *β* corresponds to biological ageing rate, and *α* to ageing-independent causes of mortality. Here, we investigate the biological basis of *α* and *β* using the nematode *Caenorhabditis elegans*, through the combined study in populations and individuals of effects of life-extending interventions on mortality and age-changes in health. We demonstrate that reductions in *β* arise not from slowed biological ageing, but rather from expansion of decrepitude (gerospan) in longer-lived population members. In contrast, reductions in *α* better reflect healthspan expansion, an indicator of slowed biological ageing. Thus, our investigation presents a new, empirical understanding of the Gompertz parameters that inverts their traditional interpretations.

## Abstract (web summary)

Zhang and Gems show that mortality rate acceleration in nematode populations reflects individual variation in the duration of late-life decline, not biological ageing rate, challenging long-standing views in the biodemography of ageing.

## Introduction

Ageing (senescence) is the main cause of disease and death in the world today^1^. Studies of the biology of ageing often employ short-lived animal models such as the nematode *C. elegans*. Though biological ageing affects individuals, researchers investigating it frequently use demographic (population) ageing as a metric^2,3^. However, it is difficult to link demographic metrics (e.g. changes in mean lifespan) to determinative biological mechanisms.

This is true of a salient and enigmatic feature of demographic ageing: the exponential increase in all-cause mortality rate during adulthood, that is common (though not universal) among animal species^4,5^. This was described in human populations two centuries ago by Benjamin Gompertz, and his eponymous equation^6,7^

1 $$\mu (x)={\alpha e}^{\beta x}$$

**Fig. 1:**
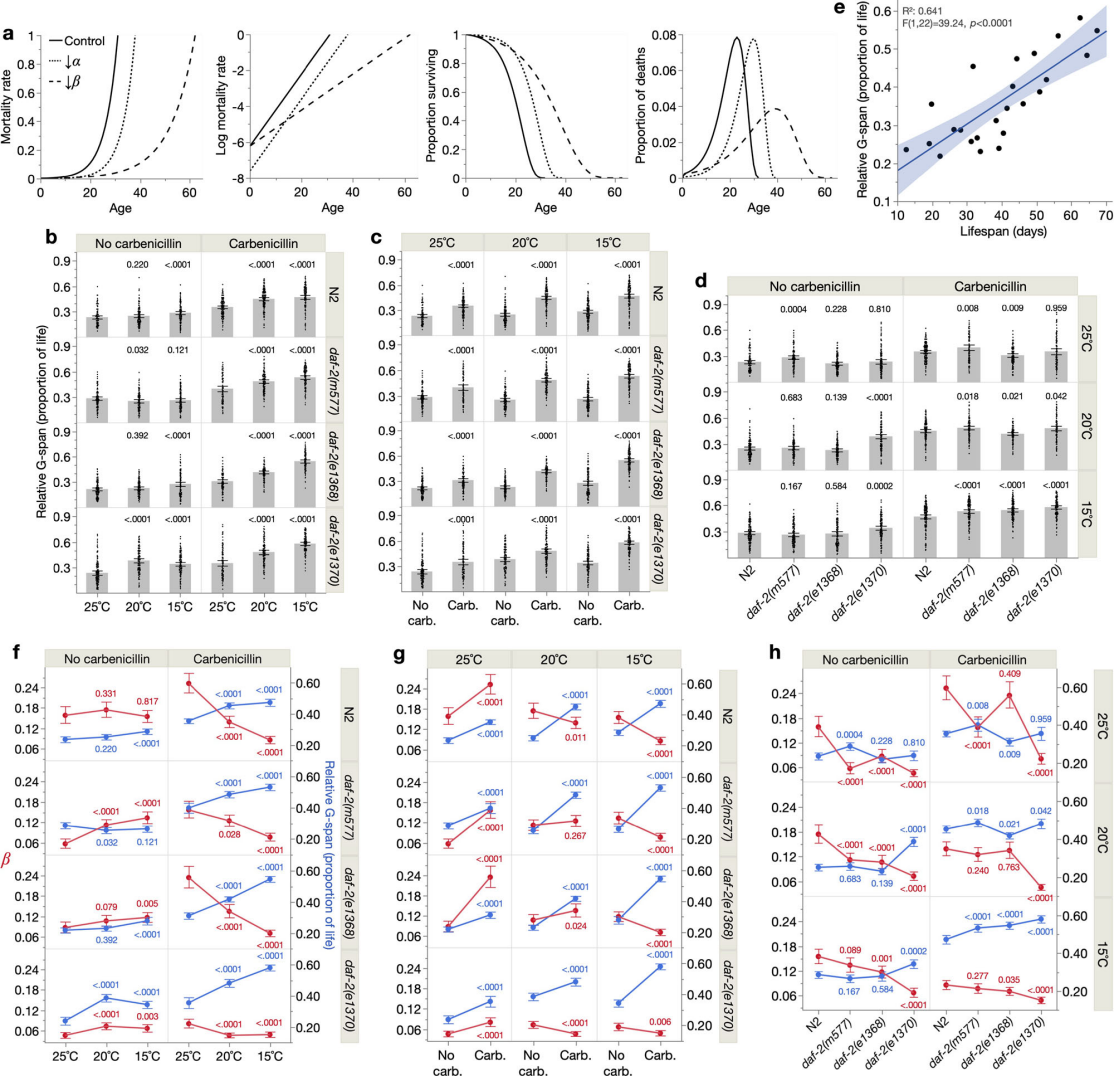
Effects of life-extending treatments on biological and demographic parameters. **a** Effects of decreasing *α* and *β* on demographic measures. Control: *α*, *β*: 0.002, 0.2; ↓*α*: *α*, *β*: 0.0005, 0.2; ↓*β*: *α*, *β*: 0.002, 0.1. First panel, effect on mortality rate. Here, *α* and *β* respectively control the scale and rate of exponential mortality rate increase. Second panel, effect on ln-transformed mortality rate. This transforms the exponential into a straight line, where *α* and *β* respectively control its intercept and gradient. Though a simple means to visualise *α* and *β* effects, this practice can be misleading and lead to improper parameter estimation^14,53–55^. Third panel, effect on survival proportion. Here, *α* and *β* approximately shift and stretch, respectively, the survival curve along the x-axis. Fourth panel, effect on mortality frequency. Here, *α* and *β* approximately shift and stretch, respectively, the mortality frequency distribution along the x-axis. That is, inter-individual lifespan variation is increased more by reductions in *β* than *α*^40^. **b**–**e** Life-extending interventions disproportionately extend gerospan (G-span). Effects of **b** reduced temperature, **c** carbenicillin, and **d** *daf-2(rf)* on mean relative gerospan (G-span^rel^). *daf-2(e1368)* was previously observed to increase G-span^rel^ at 20°C^38^; that we did not see this could reflect differences in how G-span was measured. N2, wild-type. Statistical significance of G-span^rel^ differences was assessed by two-tailed Student’s t-tests (*p* values annotated), showing 95% confidence intervals. **e** Mean G-span^rel^ is positively related to mean lifespan across the 24 cohorts, assessed by least-squares linear regression and F-test, showing 95% confidence regions. **f**–**h** Correspondence between reduced *β* and increased relative gerospan. Effects of **f** reduced temperature, **g** carbenicillin, and **h** *daf-2(rf)* on *β* and relative gerospan (G-span^rel^). G-span^rel^ values, 95% confidence intervals and statistical significance symbols are replotted from **b**–**d** and *β* calculated by maximum likelihood estimation, and statistical significance of *β* differences assessed by likelihood ratio tests, showing 95% confidence intervals. Analyses in **b**–**h** were performed on the pool of 4 biological replicates (Trials 3–6, see Supplementary Table 1 for sample sizes), with censored individuals excluded in **b**–**e** and right censored in **f**–**h**. Source data are provided as a Source Data file.

Here, mortality rate *μ(x)* at age *x* is a function of a scale parameter *α* and rate parameter *β*, with the latter specifying the mortality rate acceleration with age. Accordingly, *α* and *β* together determine survival, and mortality rate and frequency functions (Fig. 1a).

But to what do these demographic parameters correspond in terms of biological ageing, within individuals? Because *α* quantifies the initial mortality rate prior to ageing (*x* = 0, usually defined as in early adulthood), it is widely interpreted as relating to ageing-independent mortality, arising for example from extrinsic hazards or intrinsic vulnerabilities^8,9^. By contrast, *β* describes the rate of mortality rate increase, and because mortality rate increase has been traditionally defined as ageing, *β* has thus become widely interpreted as capturing the rate of ageing. Here, equivalence between demographic ageing (mortality rate increase) and biological ageing (rate of ageing) is assumed. However, these standard biological interpretations of *α* and *β* have been challenged^10–17^, and to our knowledge have yet to be empirically validated by direct comparison of demographic and biological ageing. While the relationship between lifespan and late-life health has been investigated previously, including in *C. elegans*^18–27^, the specific question of the biological basis of the Gompertz parameters has not been directly and empirically examined.

Here, we explore the biological meaning of *α* and *β* in terms of age changes in health at the individual level. To this end, we simultaneously quantified biological and demographic ageing by means of longitudinal analysis of individual *C. elegans* subjected to life-extending interventions. Specifically, we investigated: (1) how life-extension affects the duration of healthy versus decrepit life, (2) whether such changes in health/decrepitude correlate with changes in the Gompertz parameters, (3) whether these correlations can be explained causally, and (4) whether the Gompertz parameters can be causally related to specific ageing-related pathologies. We will present our results in the order of these aims. In brief, we find that *β* is a measure of inter-individual variability in gerospan (late-life decrepitude) rather than biological ageing rate, and *α* of biological ageing rate rather than ageing-independent processes.

## Results

We examined the effects on biological and demographic ageing of three types of intervention that increase *C. elegans* lifespan: (i) reduction of ambient temperature from 25°C to 20°C and 15°C, which has both thermodynamic and gene-mediated effects upon nematode ageing^2,28,29^, (ii) prevention of late-life infection (by dietary *E. coli*) using antibiotics^30^, and (iii) reduction-of-function (*rf*) mutation of the *daf-2* insulin/IGF-1 receptor^3,31^, specifically the class 1 alleles *daf-2(m577)* and *daf-2(e1368)*, and the class 2 (more pleiotropic) allele *daf-2(e1370)*.

Lifespan was measured for all 24 combinations of conditions (3 temperatures, ± carbenicillin, 4 genotypes) in 6 successive trials. To relate the Gompertz parameters to individual-level biological ageing features, in 4/6 trials, all animals were tracked individually and their locomotory health assessed every 2–3 days until death. Adapting an established protocol^32,33^, animals were scored as either youthful (healthy, sinusoidal locomotion) or decrepit (non-sinusoidal/uncoordinated locomotion or immotility) following controlled physical stimulation with a platinum wire; this induces an escape response that reveals true locomotory capacity rather than behavioural locomotory preference^19^. We will refer to absolute and relative healthspan (H-span^abs^, H-span^rel^) and gerospan (G-span^abs^, G-span^rel^) to describe, respectively, the total number of days or proportion of life spent in youthfulness or decrepitude. In other words, for every individual, H-span^abs^ + G-span^abs^ = lifespan, and H-span^rel^ + G-span^rel^ = 1. Necropsies^23^ were also performed to determine the mode of death of animals in these 4 trials.

Unless indicated otherwise, we will characterise the relationship between demographic and biological ageing using the combined pool of these 4 trials described above (*n* = 116–179/cohort), as they collected both lifespan and health data. Mean lifespan and the Gompertz parameters (calculated by maximum likelihood estimation^34^) were stable between these 4 trials (Supplementary Fig. 1a, Supplementary Table 1), and the Gompertz model provided an excellent fit to their combined pooling, predicting mean, median, and first and third quartile lifespans across the 24 cohorts with *R*^2^ = 0.98–1.00 (Supplementary Fig. 2a–b, e–h). To confirm that sample sizes are sufficient for model fitting, and that these Gompertz parameters are representative of these cohorts, we ran two additional larger trials (measuring lifespan only), increasing total pooled sample sizes by 2–3-fold to 326–396/cohort (Supplementary Table 1). Notably, the Gompertz fit still predicted the above lifespan measures, with *R*^2^ = 0.97–0.99 (Supplementary Fig. 2c–d, e–h), and the Gompertz parameters were near-identical to those from the pool of the 4 smaller trials (Supplementary Fig. 1b). Anderson-Darling goodness-of-fit tests also supported these Gompertzian fits in most cohorts (21–22/24) despite localised deviations within some (Supplementary Table 2).

These data are therefore consistent between trials and robustly fitted by the Gompertz model. Although other mortality models can better fit many *C. elegans* populations^20,35–37^, our aim is not to find the very-best-fitting model, but to understand the biological basis of the mathematically parsimonious and widely employed Gompertz model.

### i) Three life-extending intervention classes disproportionately extend gerospan

From the 24 cohorts, we focused on a total of 46 treatments of interest: 16 lower temperature treatments (20°C and 15°C, vs 25°C, across antibiotic and genotype backgrounds) + 12 antibiotic treatments (carbenicillin vs no carbenicillin, across temperature and genotype backgrounds) + 18 *daf-2(rf)* treatments (3 *daf-2(rf)* alleles vs the N2 wild-type, across temperature and antibiotic backgrounds). As expected^2,3,30^, almost all (44/46) treatments increased mean lifespan (average class effects for lower temperature, antibiotic and *daf-2(rf)* treatments: +42.0%, +58.9%, +77.1%, respectively; Supplementary Fig. 3, Supplementary Table 1).

To begin relating lifespan to the biological ageing process, we asked if these life-extending treatments affect G-span^rel^ (proportion of life in gerospan). All (12/12) carbenicillin treatments increased G-span^rel^ (Fig. 1c), in line with a prior report of antibiotic-mediated morbidity expansion^38^. Somewhat against expectation, 12/16 lower temperature treatments also increased G-span^rel^ (Fig. 1b). 9/18 *daf-2(rf)* treatments also increased G-span^rel^ while only 2/18 decreased it (Fig. 1d). This is broadly consistent with previous reports of morbidity expansion in *daf-2(rf)* mutants^16,18,38^, including the finding that this expansion results from greater resistance to life-limiting infection (by the *E. coli* food source) than wild-type animals^26,38^. Indeed, at the standard culture temperature of 20°C, *daf-2(e1370)* increased G-span^rel^, and this increase was largely suppressed by carbenicillin (Fig. 1d). However, among other *daf-2(rf)* allele/temperature combinations, G-span^rel^ patterns varied markedly, revealing more complex allelic and environmental effects of *daf-2(rf)* on the relationship between health and lifespan. This condition-dependency may have contributed to disagreements in the literature about *daf-2(rf)* effects on relative morbidity^19,26^.

Overall, the longevity interventions tested here mostly increase the proportion of life in poor (aged) health. Indeed, direct regression of mean G-span^rel^ against mean lifespan for the 24 cohorts revealed a strong positive relationship (Fig. 1e). While gerospan here is necessarily simplified to a single portion of life, its definition (non-sinusoidal locomotion or immotility, despite stimulation) captures well-characterised stages in nematode ageing and is a physiologically integrative measure of overall health^32,33^. Accordingly, the age of this gerospan onset provides one measure of the overall rate of change in health, or equivalently, a simple estimate of an individual’s overall rate of ageing. Notably, the G-span^rel^ increases observed across these life-extending treatments argue against a simple deceleration of ageing, which would be expected to stretch H-span^abs^ and G-span^abs^ proportionally, leaving G-span^rel^ unchanged.

### ii) Correspondence between reduced *β* and increased relative gerospan

Next, we considered the central question of this study: the relationship between biological and demographic ageing. To this end, we examined the relationship between effects on G-span^rel^ and the Gompertz parameters by the 46 life-extending treatments; we start with the rate parameter *β*, reduction of which is typically equated with decelerated biological ageing.

First, we considered effects of reducing temperature. On carbenicillin, lower temperature decreased *β* in all genotypes (Fig. 1f), which would be consistent with a general slowing of the rate of living. However, without carbenicillin, *β* was unchanged in wild-type and even increased in *daf-2* mutants, suggesting complex effects of genotype and temperature on host-*E. coli* interactions. Surprisingly, *β* reduction by lowered temperature (on carbenicillin) consistently co-occurred with G-span^rel^ increase (Fig. 1f, right), suggesting that *β* reduction here reflects neither simply slowed ageing rate nor improved overall quality of life.

We similarly evaluated effects of the antibiotic treatments. Against the expectation that preventing infection (a largely extrinsic insult) might reduce *α* alone, carbenicillin decreased *β* in all genotypes at 15°C, and in wild-type and *daf-2(e1370)* at 20°C (Fig. 1g). In each case, G-span^rel^ was again increased. In the remaining carbenicillin treatments, both *β* and G-span^rel^ were increased, suggesting that *β* reduction may require G-span^rel^ increase, but not vice versa.

Finally, we assessed the *daf-2(rf)* mutations. *daf-2(e1370)*, the severest (and longest-lived) allele, also lowered *β* while increasing G-span^rel^, both off and on carbenicillin, at 15°C and 20°C (Fig. 1h). At 25°C, *β* was decreased without change in G-span^rel^, as an exception to the pattern; similarly, only 3/7 class 1 *daf-2(rf)* treatments (*daf-2(m577)* and *daf-2(e1368)*) reducing *β* increased G-span^rel^. Despite these exceptions, 21/27 (78%) of all treatments that significantly reduced *β* simultaneously increased G-span^rel^. Thus, amongst these life-extending interventions, reduction of *β* largely reflects expanded decrepitude rather than a simple deceleration of biological ageing. Of course, this does not preclude occurrence of more complex forms of ageing deceleration, as demonstrated in “Results” section (iv).

### iii) Reduced *β* reflects inter-individually variable gerospan expansion

**Fig. 2:**
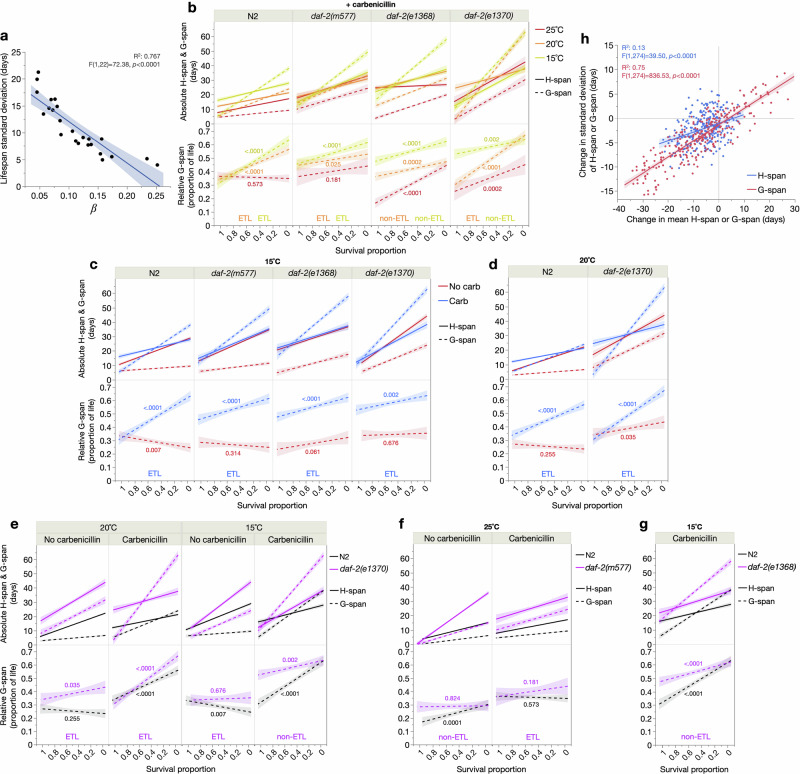
Reduced *β* reflects inter-individually variable gerospan expansion. **a** *β* is an inverse measure of lifespan standard deviation, across the 24 cohorts. **b**–**g** Effects of **b** low temperature, **c**–**d**, carbenicillin, and **e**–**g** *daf-2(rf)* on absolute healthspan and gerospan (upper panels) and relative gerospan (lower panels), plotted as linear regression fits over survival proportion (i.e. x-axis left: shorter-lived individuals, x-axis right: longer-lived individuals). In these plots, each individual is represented by two datapoints (not shown), one for H-span and one for G-span, and individuals are ordered along the x-axis by the survival proportion at their time of death (i.e. survival order). **h** Expansion of G-span is a more inter-individually variable process than expansion of H-span across all possible pairs of the 24 cohorts. In addition to a higher *R*^2^, the steeper G-span linear fit indicates that G-span variation increases faster than H-span variation under life-extending conditions. Consistent with this, the range of values for mean H-span and H-span standard deviation is less than that for G-span. All analyses in this figure are least-squares linear regressions assessed by F-tests and showing 95% confidence regions. Source data are provided as a Source Data file.

A notable feature of the demography of ageing is the high degree of inter-individual variability in lifespan. This is true of *C. elegans*, despite their being isogenic and maintained under identical conditions^39^. An often-overlooked mathematical property of the Gompertz rate parameter *β* is its inverse relation to lifespan variation, which in comparison is only marginally affected by the *α* parameter^40^ (Fig. 1a, fourth panel). Indeed, amongst our 24 treatments, *β* showed a strong inverse correlation with lifespan standard deviation (Fig. 2a).

Given this, we wondered whether this increased lifespan variation in small-*β* cohorts could result from increased inter-individual variation in the ageing process. Interestingly, it was previously estimated that G-span differences account for most (~67%) of the inter-individual lifespan variation in *C. elegans*^21^. Thus, the correlation between reduced *β* and increased G-span^rel^ in our cohorts may arise from inter-individually variable G-span expansion. To investigate this, we assessed H-span^abs^ and G-span^abs^ for all individuals in the 21 treatments displaying an inverse *β*–G-span^rel^ relationship, as explained next.

To visualise the variation in H-span^abs^ and G-span^abs^ in each cohort population, we regressed individuals’ H-span^abs^ and G-span^abs^ values over their survival proportion (Fig. 2b–g, top panels in each); here, the x-axis orders individuals by survival order (left: shorter-lived individuals, right: longer-lived individuals), therefore allowing H-span and G-span of individuals from different cohorts to be compared, akin to the y-axis of survival curves, which allows comparison of lifespans between cohorts for a given survival proportion (e.g. 0.5 for median lifespan).

We first used these plots to assess the 8 reduced temperature treatments that decreased *β* and increased G-span^rel^ (Fig. 2b). Most (5/8: 20 °C & 15 °C in wild-type and *daf-2(m577)*, and 20 °C in *daf-2(e1370)*) increased G-span^abs^ disproportionately more than H-span^abs^ in longer-lived population members (Fig. 2b top, Supplementary Fig. 4a top and middle). As a result, G-span^rel^ was increased in these longer-lived individuals (Fig. 2b bottom, Supplementary Fig. 4a bottom). This shows that life-extending interventions can act by introducing or amplifying the extended “twilight”^21^ (greater G-span^rel^) of longer-lived individuals within a population, which we will refer to as *extended twilight longevity* (ETL). A critical implication of this ETL is that the *β* reduction and associated extension of the survival curve tail arise from the disproportionate expansion of decrepitude in longer-lived population members.

Similarly, in all 6 carbenicillin treatments yielding the inverse *β*–G-span^rel^ relationship, G-span^abs^ again increased disproportionately more than H-span^abs^ in longer-lived individuals, thus increasing their G-span^rel^ (Fig. 2c–d, Supplementary Fig. 4b). Therefore, carbenicillin too is an ETL treatment that lowers *β* via variable G-span expansion. Finally, 4/7 of the *daf-2(rf)* treatments that decreased *β* and increased G-span^rel^ also did so through ETL (Fig. 2e–g, Supplementary Fig. 4c). These 4 treatments were *daf-2(e1370)* at 20°C ± carbenicillin and at 15°C without carbenicillin, and *daf-2(m577)* at 25°C with carbenicillin.

Therefore, *β* reduction reflected ETL in 5/8 reduced temperature, 6/6 antibiotic, and 4/7 *daf-2(rf)* treatments (15/21 or 71%, in total), where *β* reduction co-occurred with G-span^rel^ increase. In the 6 non-ETL exceptions (Fig. 2b, e–g), G-span^abs^ still increased mostly in longer-lived population members (i.e. reducing *β*), in particular 15°C treatment in *daf-2(e1368)* and *daf-2(e1370)* (Fig. 2b) and *daf-2(e1370)* treatment at 15°C on carbenicillin (Fig. 2e). However, this was also largely true of H-span^abs^; thus, in this minority of treatments, *β* reduction reflects inter-individually variable expansion of both G-span and H-span.

As a further test for whether *β* reduction arises from ETL, we compared contributions of H-span^abs^ and G-span^abs^ changes to *β* reduction, and to lifespan variation (standard deviation) increase (i.e. *β* reduction), in the 15 ETL treatments. Indeed, G-span^abs^ changes decreased *β* more than H-span^abs^ changes in 13/15 treatments (compared to 0/6 non-ETL exceptions) (Supplementary Table 3), and increased lifespan variation more in 14/15 interventions (compared to 3/6 non-ETL exceptions) (Supplementary Table 4). Consistent with these findings, across the 24 cohorts G-span variation increased more rapidly with mean G-span than H-span variation with mean H-span, while mean length and variability of G-span had wider ranges than that of H-span (Fig. 2h). This shows that across these cohorts, inter-individually variable expansion of G-span rather than H-span (i.e. ETL) is the primary mode of life extension.

These findings argue that expanded decrepitude not only correlates with a lower *β*, but causes it. That is, gerospan increase in longer-lived population members further increases their lifespan, thus extending the survival curve tail in the manner characteristic of a smaller *β* value (Fig. 1a, third panel). Therefore, *β* here is not a measure of intra-individual biological ageing rate as commonly understood, but rather of inter-individual heterogeneity in late-life decrepitude.

### iv) Reduced *α* reflects slowed biological ageing rate

**Fig. 3:**
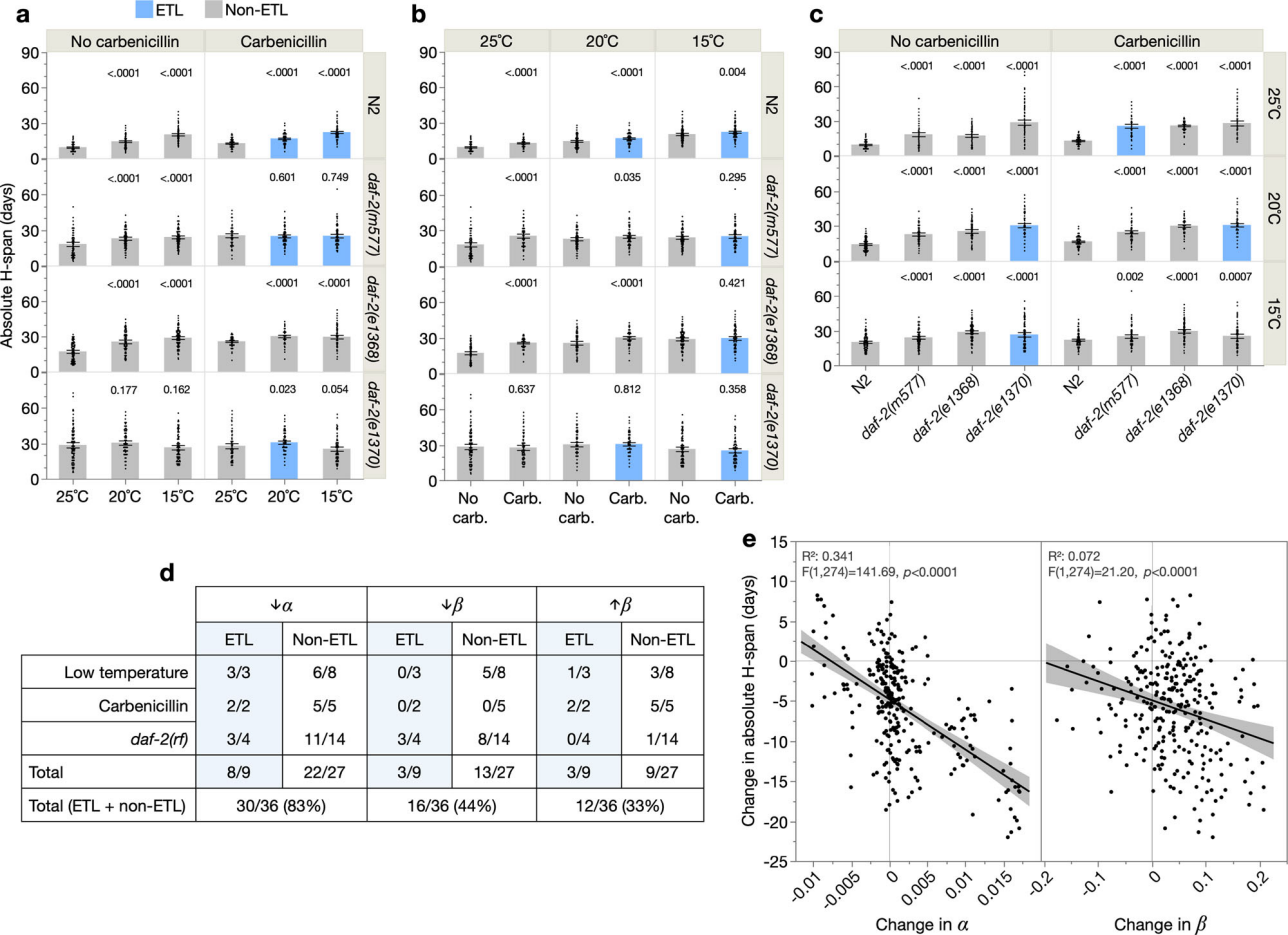
*α* outperforms *β* as a measure of absolute healthspan. Effects of **a** low temperature, **b** carbenicillin and **c** *daf-2(rf)* on mean absolute healthspan (H-span^abs^). N2, wild-type. ETL, extended twilight longevity. H-span^abs^ differences were assessed by two-tailed Student’s t-tests (*p* values annotated), showing 95% confidence intervals. This was performed on the pool of 4 biological replicates (Trials 3–6, see Supplementary Table 1 for sample sizes), with censored individuals excluded. **d** Table summarising fractions of ETL and non-ETL treatments in which H-span^abs^ increase causes the specified Gompertz parameter change, for each life-extending treatment class. **e** Least-squares linear regressions of the changes in H-span^abs^ between all possible pairs of the 24 cohorts, over the corresponding change in *α* or *β* for those pairs. The relationships were assessed by F-tests, showing the 95% confidence region. Source data are provided as a Source Data file.

Because healthspan expansion may be considered a reliable indicator of slowed biological ageing (given more time to G-span onset), we wondered if *β* reduction could at least indirectly reflect decelerated ageing, should G-span^abs^ and H-span^abs^ expansions occur together. Indeed, mean H-span^abs^ was increased in 9/15 ETL treatments (Fig. 3a–c). We therefore examined the effect of population-wide H-span^abs^ expansion on the Gompertz parameters in these 9 treatments. H-span^abs^ expansion significantly decreased *β* in 4/9 treatments but, strikingly, significantly decreased *α* in 8/9 (Fig. 3d, Supplementary Table 5a–c). This suggests that *α*, though not traditionally equated with biological ageing, may here be a better measure of it than *β*.

To assess if this unexpected finding is idiosyncratic to ETL, we also examined the non-ETL treatments, of which 27/31 significantly extended H-span^abs^ (Fig. 3a–c). However, again, H-span^abs^ expansion decreased *α* in 22/27 (81%) of these non-ETL treatments, but decreased *β* in only 13/27 (48%) (Fig. 3d, Supplementary Tables 5a–c). Additionally, H-span^abs^ expansion *increased β* in 9/27 treatments, including in all carbenicillin treatments; thus, slowing biological ageing can even increase *β*. Considering all treatments (ETL plus non-ETL), H-span^abs^ expansion decreased *α* in 30/36 (83%) cases, decreased *β* in 16/36 (44%), and increased *β* in 12/36 (33%). This suggests that *α* should better predict H-span^abs^ than *β* amongst the 24 cohorts, and this indeed proved to be the case (Fig. 3e).

In summary, healthspan expansion, a plausible metric of slowed biological ageing, more consistently reduces *α* than *β*, and even increases *β*. Therefore, overall, our empirically-based findings invert traditional interpretations of the biological meaning of the two Gompertz parameters.

### v) *α* and *β* describe inter-individual heterogeneity in age-related infection

**Fig. 4:**
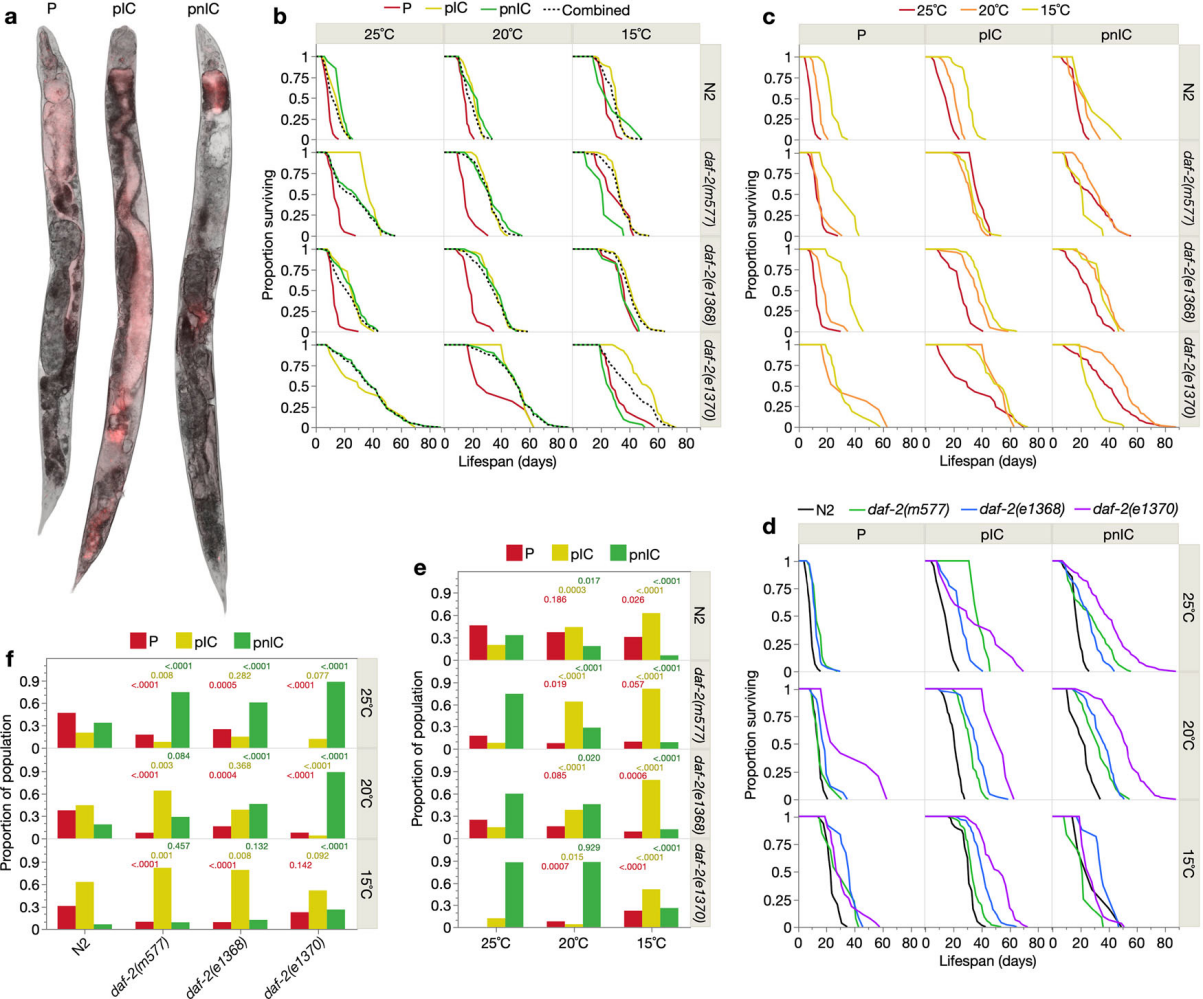
Life-extending interventions change lifespan and prevalence of pathological subpopulations. **a** Images of representative P, pIC, and pnIC corpses, fed throughout life with RFP-expressing *E. coli*; overlay of brightfield and epifluorescence images. **b** Survival curves of P, pIC and pnIC subpopulations and whole population (“Combined”). **c**–**d** Effect of **c** low temperature and **d** *daf-2(rf)* treatments on subpopulation lifespan. Log-rank *p* values of depicted comparisons for (**b**–**d**) are presented in Supplementary Table 6. Survival proportions were obtained from Kaplan-Meier lifespan analysis using pseudofrequencies (in place of frequency) to account for censors (see Methods for details). **e**–**f** Effects of **e** low temperature and **f** *daf-2(rf)* on subpopulation prevalence. Differences in prevalence were assessed by Pearson’s chi-squared test (*p* values annotated). This was performed on the pool of 3–4 biological replicates (Trials 4–6 for N2 cohorts and Trials 3–6 for all other cohorts, see Supplementary Table 1 for sample sizes), with censored individuals excluded. At 25 °C no *daf-2(e1370)* P deaths were observed, as previously noted^41^. Source data are provided as a Source Data file.

To further understand the biological mechanisms underpinning the Gompertz parameters, we performed necropsies on all corpses, building upon established mortality deconvolution methodology^23^. We scored for ageing-related bacterial colonisation of the pharynx and intestine (Fig. 4a, Supplementary Fig. 5a–b; in non-carbenicillin cohorts). We noted that almost all corpses with a swollen, infected pharynx (P or “big P”, as opposed to p or “small p” with an atrophied, uninfected pharynx)^23^ also had intestinal colonisation by *E. coli*, but not vice versa. Thus, P corpses are largely a subset of those with intestinal colonisation (Supplementary Fig. 5c). Death type was accordingly used to define three biologically-distinct subpopulations: P, pIC (p with intestinal colonisation), and pnIC (p with no intestinal colonisation) (Fig. 4a, Supplementary Fig. 5c). Consistent with prior findings^23^, lifespan was shorter for P than p (pIC and pnIC) subpopulations for most treatments (Fig. 4b, Supplementary Table 6). Lifespan was also shorter for pIC than pnIC populations (at 15°C and 25°C, but not 20°C). Thus, the three subpopulations exhibit distinct ageing trajectories.

We then asked how this inter-subpopulation heterogeneity affects *β*. Notably, *β* was consistently smaller in whole populations than in subpopulations (Supplementary Table 7). This provides empirical evidence of how *β* can underestimate demographic ageing rate in the presence of hidden subpopulation heterogeneity^11^. Moreover, the traditional interpretation of *β* would paradoxically predict that subpopulations age biologically faster than their combination (whole population). This implies that even for isogenic individuals in identical conditions, *β* can be a measure of inter-individual heterogeneity in ageing, rather than of biological ageing rate itself.

We next examined how life-extending treatments (here, low temperature and *daf-2(rf)*, without carbenicillin) affect subpopulation prevalence and lifespan. While both treatment classes increased lifespan of most subpopulations, increases were generally greater for P by low temperature, and pIC by *daf-2(rf)* (Fig. 4c–d; Supplementary Table 6). Lower temperature modestly decreased P prevalence in all genotypes except *daf-2(e1370)*, suggesting reduced bacterial pathogenicity and/or enhanced host immunity, and allele-specific temperature sensitivity in *daf-2(e1370)*^41^ (Fig. 4e). Interestingly, however, low temperature strongly increased pIC prevalence (and thereby decreasing pnIC prevalence). Thus, reducing temperature acts antagonistically on terminal infection, respectively decreasing and increasing pharyngeal and intestinal colonisation by pathogenic *E. coli*. Meanwhile, *daf-2(rf)* treatment also decreased P prevalence as previously seen^41^ and affected pIC prevalence in an allele- and temperature-specific manner (Fig. 4f), largely consistent with known *daf-2(rf)* infection resistance^38^.

**Fig. 5:**
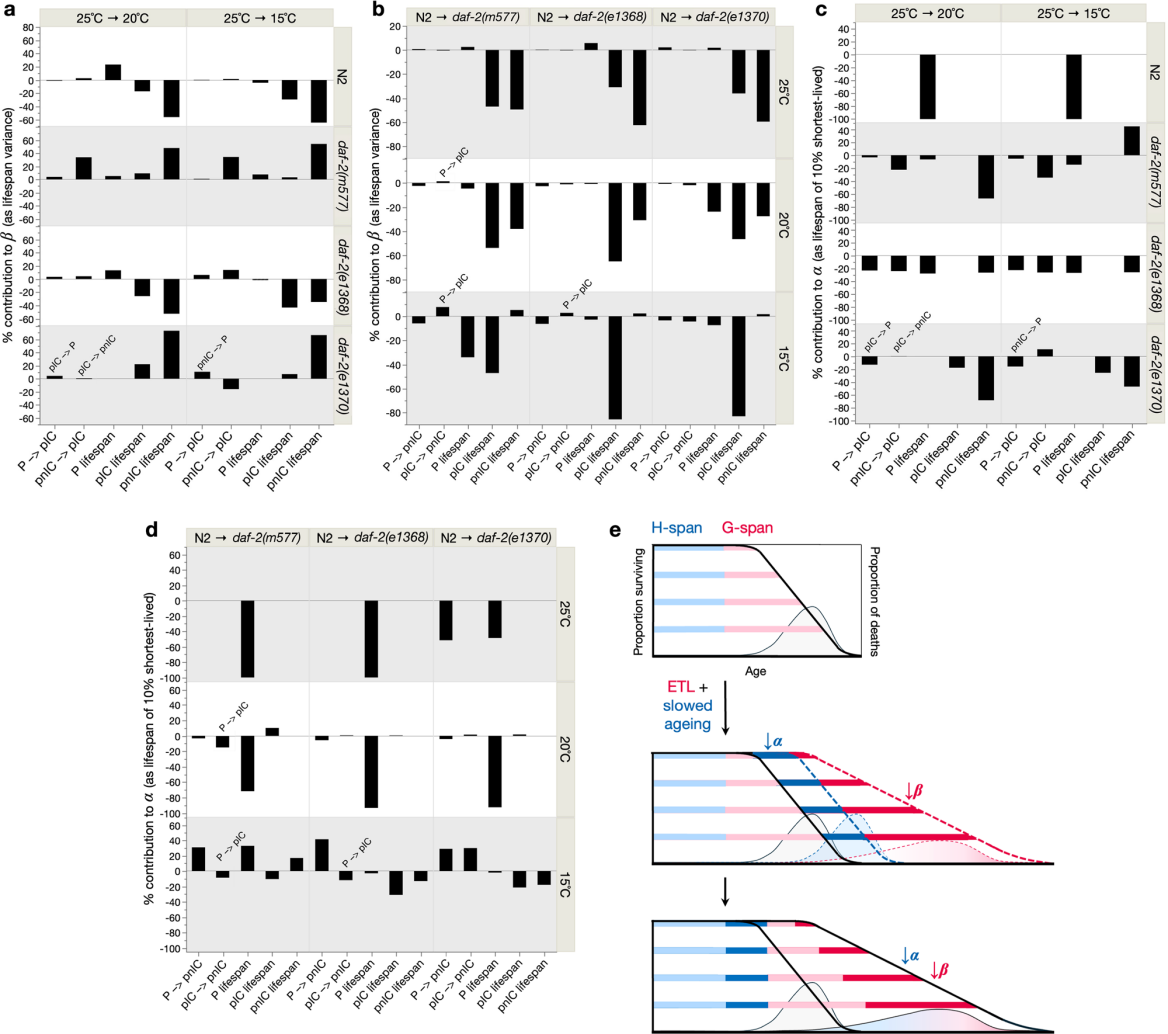
*α* and *β* reflect subpopulation-specific changes in lifespan and prevalence. Relative contributions (sum to 100%) of changes in subpopulation prevalence and lifespan to overall change in population (**a**–**b**) *β* and (**c**–**d**) *α* in (**a**, **c**), low temperature and (**b**, **d**), *daf-2(rf)* treatments. Bar height indicates contribution magnitude (%) and bar direction (+ or –) indicates contribution direction (increase or decrease Gompertz parameter). The first two x-axis items are component contributions of changes in subpopulation prevalence (based on principles of parsimony, Fig. 4e–f), while the remaining x-axis items are contributions of changes in subpopulation lifespan. Overlaid angled labels reflect different subpopulation prevalence changes for those specific treatments. See Methods for full analysis details. Source data are provided as a Source Data file. **e** Summary schematic of the biological basis of the Gompertz parameters across our experimental cohorts, depicting a hypothetical intervention that reduces both *α* and *β*. Each panel depicts healthspan (H-span; blue bar segment) and gerospan (G-span, red bar segment) for four individuals representative of their depicted lifespan within the population (age at end of bar), as bordered by the survival curves (left y-axis, bold data lines). *α* reduction arises from H-span expansion (equally across individuals in this simplified depiction), causing the approximately parallel right-shift of the survival curve (middle panel, dashed blue) that occurs in *α* reduction. *β* reduction arises from inter-individually variable G-span expansion (greater in longer-lived individuals), causing the approximate horizontal stretch of the survival curve (middle panel, dashed red) that occurs in *β* reduction. Bottom panel: overall effect of this hypothetical intervention with H-span and G-span arranged in order. In all panels, probability distributions of death times corresponding to the survival curves are overlaid (right y-axis, thin data lines), showing that *α* reduction causes an approximate right-shift of death ages, whereas *β* reduction causes an additional stretch that increases lifespan variation more. The area under these probability distributions is shaded to reflect the variation in H-span/G-span between different population members: *α* reduction involves similar expansion of H-span in all individuals (solid blue shading), whereas *β* reduction involves greater G-span expansion in longer-lived individuals (red gradient shading). Therefore, *β* describes the degree of inter-individual variation in health and lifespan, and not biological ageing rate, which is better captured by *α* as H-span length.

Finally, we determined the relative contribution of these changes in subpopulation lifespan and prevalence to the Gompertz parameters. Effects of low temperature on *β* proved to be determined mainly by changes in pnIC lifespan, followed closely by the increase in pIC to pnIC ratio in *daf-2(m577)* (Fig. 5a, Supplementary Fig. 6a). In *daf-2(rf)* treatment, changes in *β* were again primarily determined by changes in pnIC and/or pIC lifespan, rather than in P lifespan or subpopulation prevalence (Fig. 5b, Supplementary Fig. 6b). Thus, *β* is largely a function of p lifespan; specifically, increases and decreases in *β* typically result, respectively, from decreased and increased p (pIC and/or pnIC) longevity. This further demonstrates that *β* is less a measure of biological ageing rate than inter-individual heterogeneity, here as subpopulation-specific responses to longevity interventions.

We similarly deconvolved determinants of *α*. In low temperature treatment, determinants of *α* were genotype-specific: *α* was wholly determined by P lifespan in wild-type, mainly by pnIC lifespan in *daf-2(m577)* and *daf-2(e1370)*, and by all determinants except pIC lifespan in *daf-2(e1368)* (Fig. 5c, Supplementary Fig. 6a). In *daf-2(rf)* treatment, *α* was primarily determined by P lifespan at 25 °C and 20 °C, while at 15 °C, several determinants were important; in common, the increase in pnIC to P ratio (Fig. 5d, Supplementary Fig. 6b). Therefore, unlike *β*, *α* is largely a function of P lifespan and subpopulation prevalence, but like *β*, reflects subpopulation-specific responses to life-extending interventions.

Taken together, these findings provide further evidence that being demographic measures, the Gompertz parameters can often reflect inter-individually variable biological changes whose complexity is unlikely to be captured by the traditional theoretical interpretations of the parameters.

## Discussion

This study investigates the biological underpinnings of Gompertzian demographic ageing in *C. elegans* under 46 life-extending conditions. It demonstrates how the Gompertz rate parameter *β* better corresponds to inter-individual gerospan variation than biological ageing rate, while the Gompertz scale parameter *α* better reflects biological ageing rate than ageing-independent determinants of mortality (Fig. 5e). Additionally, both parameters are the product of subpopulation-specificity in patterns of late-life disease. These findings underscore the great tenuousness of the correspondence between parameters describing population ageing on the one hand, and the biological ageing process of component individuals on the other^11^.

We have shown how reduction of *β*, usually interpreted as slowed biological ageing, can reflect greater gerospan expansion in longer-lived individuals, which extends the survival curve tail and increases lifespan variation. Consequently, longer-lived population members experience extended twilights (i.e. longer relative gerospans), as previously described^21,22,26^. One study reported the opposite^25^: shorter relative gerospans in longer-lived population members; one explanation could be methodological differences, such as the automated measurement of spontaneous (rather than stimulated) movement and use of floxuridine to inhibit progeny production, which has known effects on nematode ageing and lifespan^42–44^.

In our study, we describe how the extended twilight occurs also *between* populations in response to life-extending treatments, which we termed *extended twilight longevity* (ETL). Thus, here *β* reflects not biological ageing rate in the usual sense, but gerospan variability in ETL (Fig. 5e). Such ETL could potentially contribute to the temporal scaling of survival curves previously described in *C. elegans*^20^. Notably, our reinterpretation of *β* differs from that of existing heterogeneity-based models, e.g., Vaupel et al.^9^, which assume that at least within subpopulations, *β* does reflect biological ageing rate. In contrast, our data suggest that *β* better reflects heterogeneity than biological ageing rate between *and* within subpopulations.

Given the gradual nature of ageing, any delineation of healthspan and gerospan is inevitably somewhat arbitrary^21,24^. It is after the highly coordinated stages of development and reproduction that ageing begins and consequent inter-individual variation in age decline emerges, in midlife in nematodes by one estimate^45^. Thus, it is likely that the contribution of gerospan variation to *β* has even been underestimated, given that gerospan, as we defined it, excluded earlier ages with subtler senescent changes.

Regarding the Gompertz scale parameter *α*, past attempts to define its biological meaning include intrinsic, ageing-independent “vulnerability” or “frailty”, and extrinsic, age-related hazards, but not biological ageing rate itself^4,8,9,14^. We have shown that *α* outperforms *β* as a predictor of healthspan length, arguing against the “not ageing” interpretation, and providing experimental support for earlier doubts about the above traditional views of *α*^13,14,46^ (Fig. 5e).

Intriguingly, a recent study^27^ described a mathematical model of the ageing process that predicts a compression of relative gerospan by life-extending treatments that increase the *relative* steepness of the survival curve, and proportional scaling (no change) or even expansion of relative G-span by treatments that maintain this relative steepness. Translating these survival curve effects into approximate Gompertz terms reveals a close concordance between our two studies: parallel shifts or rectangularisation of the survival curve (i.e. involving *α* reduction) involve healthspan expansion, while horizontal stretching of the survival curve (i.e. involving *β* reduction) involves more gerospan expansion. Furthermore, the authors supported their theoretical predictions with mined, population-average health and mortality data from nematodes, fruit flies and mice^27^. These data thus provide independent support for our findings and extend their potential relevance to other species. Our study further extends this through individual-level analyses of the relationship between health/gerospan and lifespan, which is required to explain survival curve shape in terms of the inter-individual distribution of biological ageing profiles.

Are our nematode-derived reinterpretations of *α* and *β* likely to be applicable to higher animals? The aforementioned study^27^ and the frequent occurrence of Gompertzian ageing throughout the animal kingdom^4,5^ at least suggests this possibility. Like interventions that extend lifespan in *C. elegans*, the evolution of longer-lived mammals from shorter-lived ones often involves coupled reductions in both *α* and *β*, where mortality is both postponed and spread out over greater lengths of time. For instance, *α* at puberty decreases from 0.03 in laboratory mice to 0.0002 in humans, and subsequent mortality rate doubling time (an inverse measure of *β*) respectively increases from 0.27 to 8 years^4^.

This raises the interesting question of whether evolution of greater longevity might also reduce *α* by extending healthspan and reduce *β* by ETL (Supplementary Fig. 8). In other words, the reduction of *β* in longer-lived mammals might only indirectly reflect reduced biological ageing rate, which is instead directly reflected in the reduction of *α*. This coupled evolution of healthspan and gerospan expansion, and therefore *α* and *β*, could emerge from biological constraints present between mechanisms of development and ageing^47^ that exhibit evolutionary conservation. This would be consistent with the proportional scaling between life stages across mammalian species, such as that between ontogenetic span and adult lifespan ( ~ 1:4)^48^. Additionally, the greater variability of gerospan than healthspan could arise from the late-life natural selection shadow, a key determinant of the evolution of ageing^49,50^ that predicts greater optimisation (thus, standardisation) of early than later-life traits (Supplementary Fig. 8b).

In summary, our findings invert traditional interpretations relating to Gompertzian population ageing, in demonstrating that the rate parameter *β* can reflect not biological ageing rate but inter-individual variation in gerospan, that the scale parameter *α* can reflect less ageing-independent mechanisms than ageing rate itself, and that both parameters reflect subpopulation-specific rather than population-wide traits. The approach used here, the combined analysis of individual and population ageing, should prove similarly informative for understanding the biology of mortality patterns in higher organisms.

## Methods

### *C. elegans* culture and strains

*C. elegans* were maintained at 20 °C using standard protocols^51^, on Nematode Growth Medium (NGM) plates seeded 2 days before use with a bacterial food source (*Escherichia coli* OP50). Floxuridine (5-fluoro-2-deoxyuridine), sometimes used to block progeny production, was *not* used in this study. Nematode strains used were: N2 (wild-type, hermaphrodite stock^52^), GA1959 *daf-2(m577) III*, GA1960 *daf-2(e1368) III*, and GA1928 *daf-2(e1370) III*. All strains were raised from egg at 20 °C on live *E. coli*, and transferred at L4 stage to the appropriate experimental conditions (15 °C, 20 °C, or 25 °C; with or without carbenicillin). Carbenicillin solution was added topically to plates one day before adding animals (further details below).

### Lifespan-only trials (Trials 1–2)

Nematodes were cultured throughout life in 60 mm Petri dishes (containing 10 mL of NGM) seeded with approximately 80 μL of *E. coli*, and where relevant, treated with 80 μL of 500 mM carbenicillin (Fisher Scientific Ltd, catalogue no. 12737149). At L4 stage (time 0 in all analyses), 30–40 animals were placed on each plate, with three plates per condition. Animals were transferred every 2 days during the reproductive period, and approximately every seven days thereafter. Scoring of survival was performed every two days. Animals showing no movement were gently touched with a platinum wire (worm pick) on the head and/or tail; those that showed no movement at all in response were scored as dead. Animals that died due to desiccation on the Petri dish wall, internal hatching of larvae, or rupture of internal tissues through the vulva, or that became contaminated by non-*E. coli* bacteria or fungi, or could not be found, were censored.

### Lifespan and healthspan trials (Trials 3–6)

Prior to the end of egg laying, nematodes were handled as for the lifespan-only trials, but with two plates containing 25 animals each, per condition. Following the end of egg laying, animals were transferred to individual wells of 24-well tissue culture plates, containing 2 mL of NGM and seeded with 3.5 μL of *E. coli* OP50, and where relevant, treated with 16 μL of 500 mM carbenicillin. Animals were subsequently transferred to fresh plates monthly, before media desiccation (plates were sealed with parafilm to delay desiccation, and to prevent bacterial/fungal contamination). Scoring of survival and censors were performed as above every 2–3 days, alongside scoring of locomotory class, and necropsy at death.

### Quantification of locomotory decline with age

Locomotory health class (belonging to H-span or G-span) was scored by classifying individuals into one of three classes, adapted from earlier systems^32,33^: A—sinusoidal locomotion; B—non-sinusoidal locomotion; C—no locomotion. To accurately determine locomotory class, animals were gently touched on the tail with a platinum wire worm pick for up to 20 s to induce an escape response that reveals movement capacity, and additionally on the head as a final check. The duration spent in A class was defined as H-span, and the summed duration spent in B and C classes as G-span. Here, B and C classes were summed to improve data tractability and to provide a definition of G-span that captures both early and late-stage functional declines.

### Necropsy analysis

Necropsy to define patterns of *E. coli*-associated pathology was performed by examining fresh corpses under a Leica MZ8 stereomicroscope (50x magnification). Scoring of swollen, bacterially-infected pharynxes (P), and uninfected, atrophied pharynxes (p) was performed as previously described^23^. Intestinal colonisation (IC) with *E. coli* was scored, where severe bacterial accumulation was observed in the anterior and/or posterior intestine. Such colonisation presented as extreme lumenal distension by proliferating bacteria and/or colonisation of the intestine beyond the lumenal barrier, with concomitant intestinal tissue degeneration and atrophy. Consistent across pharyngeal and intestinal tissues, sites of bacterial colonisation exhibit a yellowish-brown colour (as that of the *E. coli* lawn and colocalising with RFP-labelled *E. coli*), translucent and uniform texture (loss of healthy tissue structures that otherwise appear dark, granular and opaque), and swollen/distended morphology (extensive proliferation of live *E. coli*). Images of representative examples of the three corpse subpopulations (P, pIC and pnIC) are presented in Fig. 4a and Supplementary Fig. 5b.

### Microscopy

Microscopy slides were prepared by placing individual nematode corpses in a small drop of M9 buffer on 2% agar pads, under glass coverslips. Brightfield images were captured using an ApoTome.2 Zeiss microscope with a Hamamatsu digital camera C13440 ORCA-Flash4.0 V3 and Zen software, at 100x total magnification, with 125 ms exposure time and 1.1 V illumination intensity. The presence of *E. coli* OP50-RFP in the pharynx was assayed using the mRF12 channel (excitation: 577–604 nm; emission: 612 nm) at the same magnification, with 750 ms exposure time and 75% LED intensity. Brightness and contrast were adjusted equally across the entire image, and where applicable, applied equally to controls. Brightfield and RFP epifluorescence necropsy images were overlaid in ImageJ and backgrounds removed with Adobe Express (online tool). The maximum intensity threshold of RFP channel images was adjusted in ImageJ from 255 to 70 for all images.

### Mortality deconvolution (subpopulation) analyses

Age-specific survival proportions for full (not deconvolved into subpopulations) populations were obtained by conventional Kaplan-Meier analysis (including censors), from which age-specific mortality pseudofrequencies (which sum to 1) were calculated. For each age, these pseudofrequencies were partitioned into subpopulation pseudofrequencies, weighted by the proportion of total mortality at that age belonging to each subpopulation. Standard survival and mortality analyses of subpopulations were then performed utilising these pseudofrequencies (in place of conventional mortality frequency), to enable unbiased inclusion of censor data in subpopulation analyses. Survival analyses in Fig. 4b–d were performed in this manner. Contributions of subpopulation changes to the Gompertz parameters in Fig. 5a–d were similarly performed on simulated (mortality pseudofrequency-derived) survival data. Simulated survival data was generated for each component change (in subpopulation prevalence or lifespan), by accordingly combining simulated control and treatment subpopulations. Specifically, changes in subpopulation prevalence were simulated by combining treatment cohort subpopulation prevalence with control subpopulation lifespans (based on principles of parsimony, Fig. 4e–f), and changes in lifespan were simulated by combining treatment cohort subpopulation lifespan with control subpopulation prevalence, for each subpopulation at a time. The sum of these component changes closely predicts the true change between control and treatment cohorts (Supplementary Fig. 7a). Contributions of component changes to *α* and *β* were then estimated as the change in, respectively, lifespan of the 10% shortest-lived individuals and lifespan variance, which strongly predict *α* and *β* across these 12 non-antibiotic cohorts (Supplementary Fig. 7b–c) and whose component sum of these changes predicts their true change between control and treatment cohorts (Supplementary Fig. 7d–e).

### Statistics, software and data handling

Statistical tests were performed using JMP Pro (SAS Institute, Inc.), except for Gompertz parameter estimation and assessment of statistical differences between them, which were performed using WinModest^34^. Right censors were included in all Kaplan-Meier and WinModest analyses. Specific statistical tests and associated methodological details are described in the respective figure/table captions. The statistical significance threshold used in this study is *α* = 0.05. Necropsy image capture, processing and editing were performed using Zen software and ImageJ.

Analyses of treatment effects on lifespan (Supplementary Fig. 3, Supplementary Table 1) were performed on the pool of all 6 trials, while analyses relating to locomotory healthspan and gerospan (Fig. 1–3, Supplementary Fig. 4, Supplementary Tables 3–5) were performed on the pool of 4 trials (Trials 3–6; see Methods above), in which locomotory senescence was quantified. Subpopulation analyses utilising necropsy data (Figs. 4b–f, 5a–d, Supplementary Figs. 5a, 6–7, Supplementary Tables 6–7) were similarly performed on this pool of 4 trials (or 3 trials, Trials 4–6, for N2 cohorts), in which necropsy was performed. Further information and summary statistics for individual trials are provided in Supplementary Table 1, and analyses confirming the statistical suitability of these trial poolings and the Gompertz fit are provided in Supplementary Figs. 1–2 and Supplementary Table 2.

### Reporting summary

Further information on research design is available in the Nature Portfolio Reporting Summary linked to this article.

## Supplementary information

Supplementary Information

Reporting Summary

Transparent Peer Review file

## Source data

Source Data

## Data Availability

Data generated in this study on the lifespan, locomotory healthspan and gerospan, and necropsy subpopulation type of all individuals from the 6 trials (*n* = 8830), where measured, are available in the Source Data file. Source data are provided with this paper.
