# Supplementary material for: Slowed Gompertzian ageing in long-lived C. elegans results from expansion of decrepitude, not decelerated ageing
Source: Nat Commun. 2026 Apr 13;17:5182. doi: 10.1038/s41467-026-71780-7 (PMC13254367; doi:10.1038/s41467-026-71780-7)
Supplement: Supplementary file 1 — Supplementary Information [file 41467_2026_71780_MOESM1_ESM.pdf]

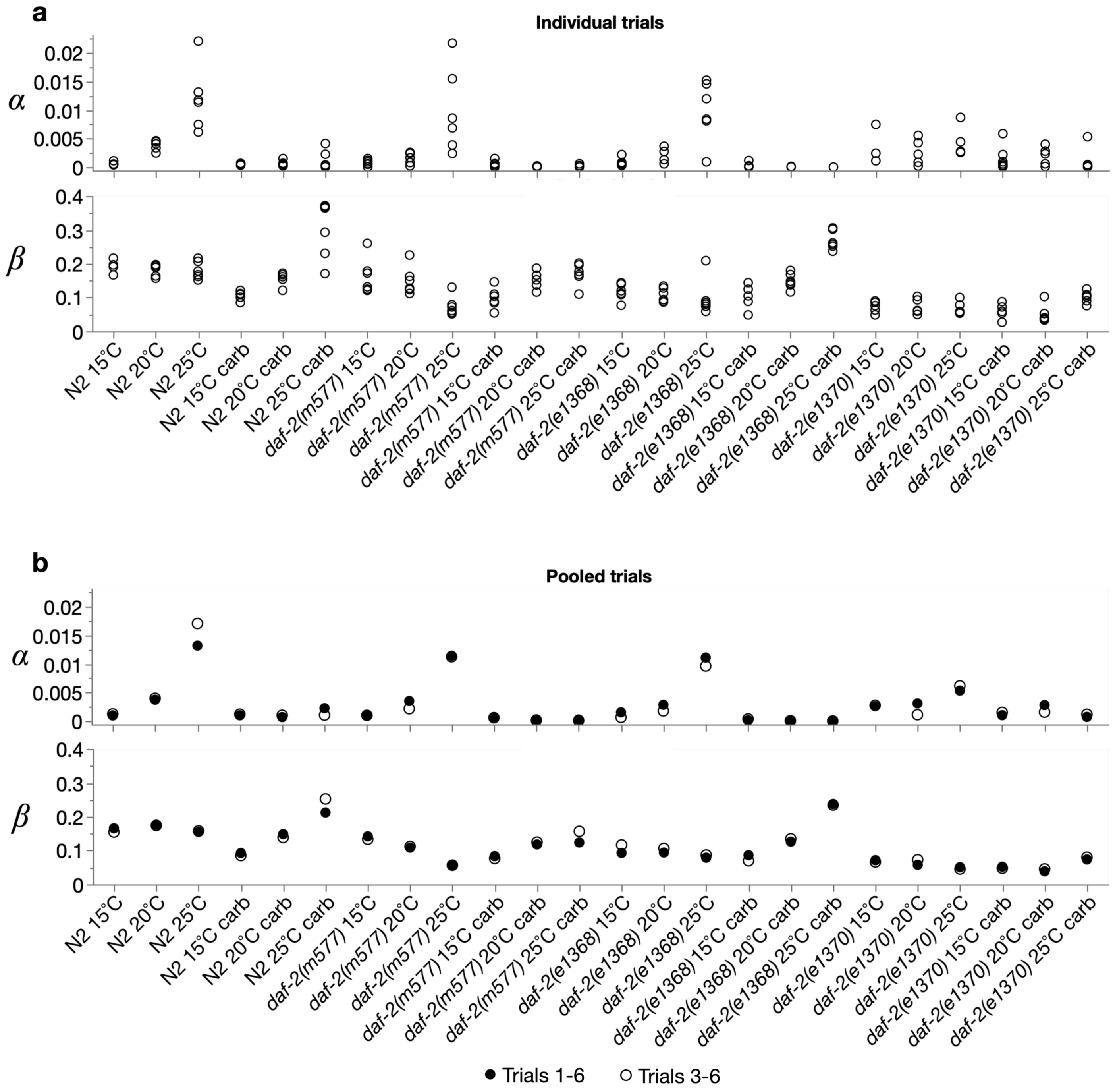

Supplementary Fig. 1 | The Gompertz parameters are stable between trials. The Gompertz parameters for each of the (**a**) individual 6 trials and (**b**) combined pool of Trials 1–6 or 3–6, for each of the 24 cohorts. Trials 3-6 (n=116–179/cohort) are those in which both lifespan and health were measured, while Trials 1–6 (n=326–396/cohort) include 2 additional larger trials in which only lifespan was measured. All primary analyses in this study compare mortality and health data from the pool of Trials 3–6. Statistical details of each trial are provided in Supplementary Table 1.

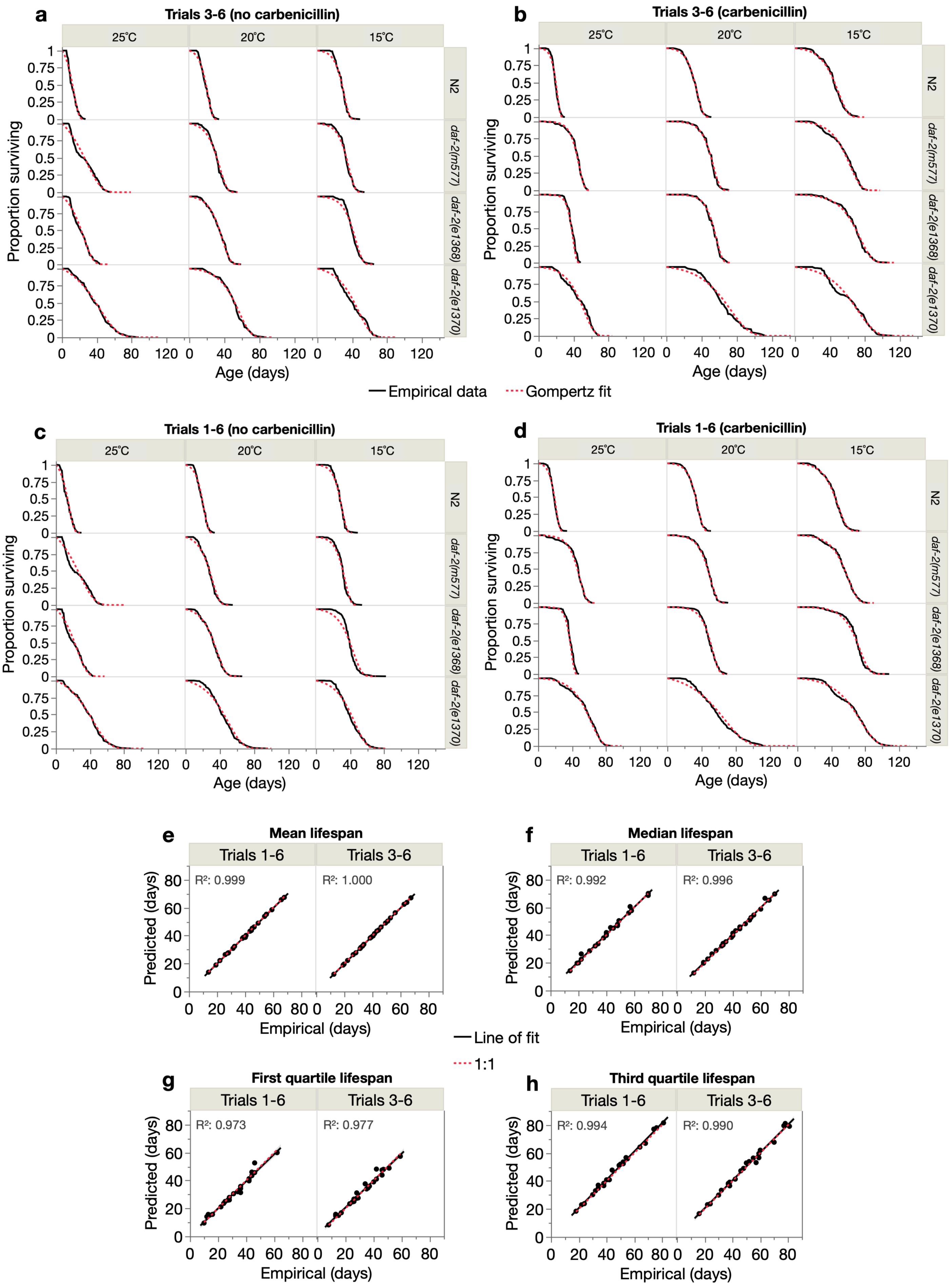

Supplementary Fig. 2 | The Gompertz model fits the data closely. (**a**–**d**) Overlay of empirical and idealised Gompertz curves for each of the 24 cohorts, for the pool of Trials 3–6 (**a**: non-carbenicillin cohorts, **b**: carbenicillin cohorts) and pool of Trials 4–6 (**c**: non-carbenicillin cohorts, **d**: carbenicillin cohorts). (**e**–**h**)

Linear regressions between true (empirical) and predicted (from the Gompertz fit) lifespan measures, across the 24 cohorts. First quartile, median and third quartile lifespans are the ages at which survival proportion equals, respectively, 0.75, 0.5 and 0.25; the strong regression relationships for each of these indicates a robust prediction of overall survival curve shape by the Gompertz model for these 24 cohorts. 95% confidence regions around the regression fits are shaded (barely visible). Trials 3-6 (n=116–179/cohort) are those in which both lifespan and health were measured, while Trials 1–6 (n=326–396/cohort) include 2 additional larger trials in which only lifespan was measured. All primary analyses in this study compare mortality and health data from the pool of Trials 3–6. Statistical details of each trial are provided in Supplementary Table 1.

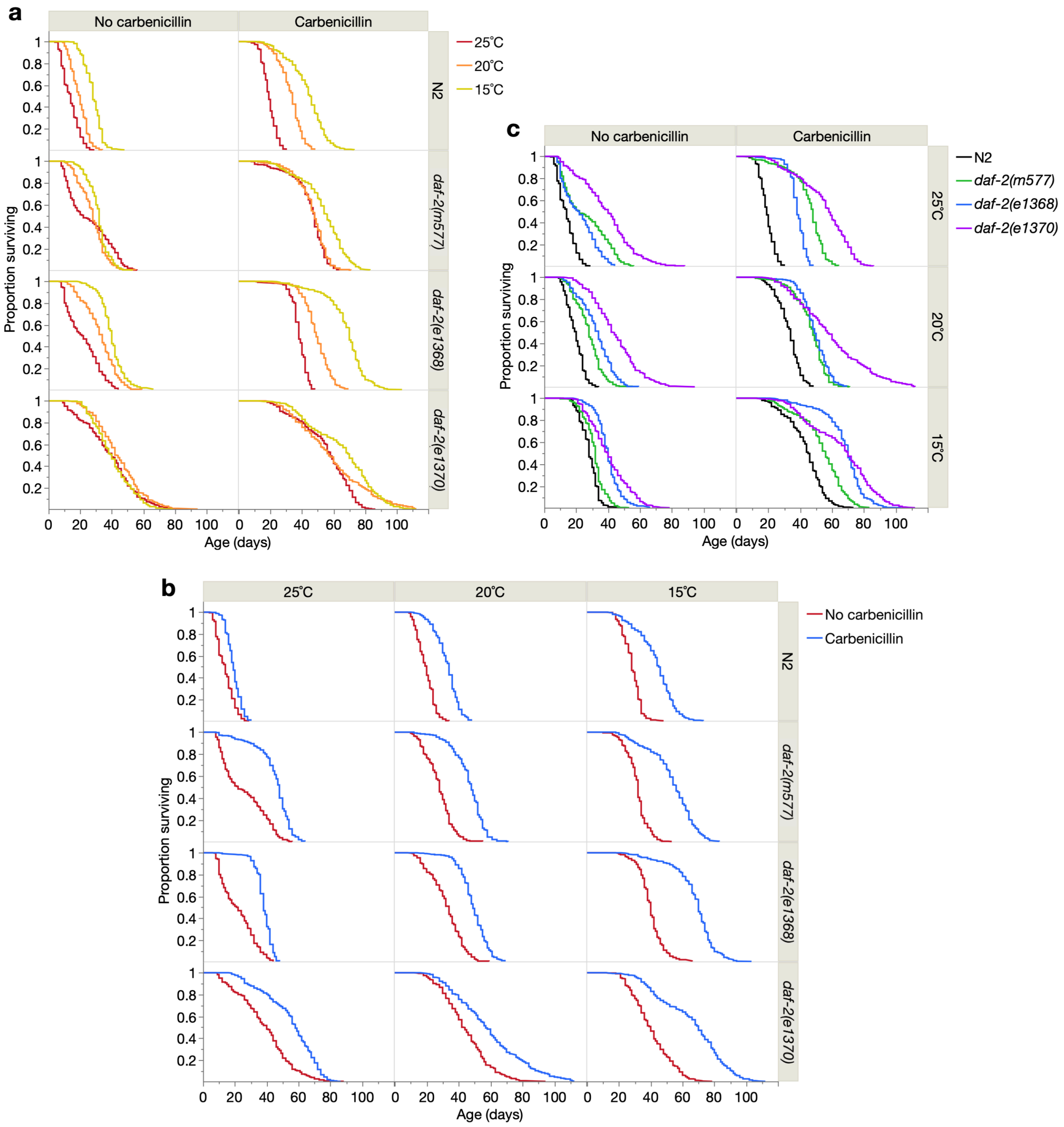

Supplementary Fig. 3 | Kaplan-Meier survival curves for all cohorts. Effects of **a**, reducing temperature, **b**, antibiotic treatment, and **c**, *daf-2(rf)*. N2, wild-type. Pool of all 6 trials. Statistical comparisons (*p* values, log rank test) are presented in Supplementary Table 1. A previous study found that culture of *daf-2(e1370)* on a different antibiotic (gentamycin) did not increase lifespan, and even slightly shortened it, suggesting that this mutation might confer full resistance to life-shortening effects of live *E. coli*[1]. Our findings using carbenicillin (**b**) imply that all 3 *daf-2* mutants succumb to effects of proliferating *E. coli* in later life, likely reflecting inevitable, eventual immunosenescence.

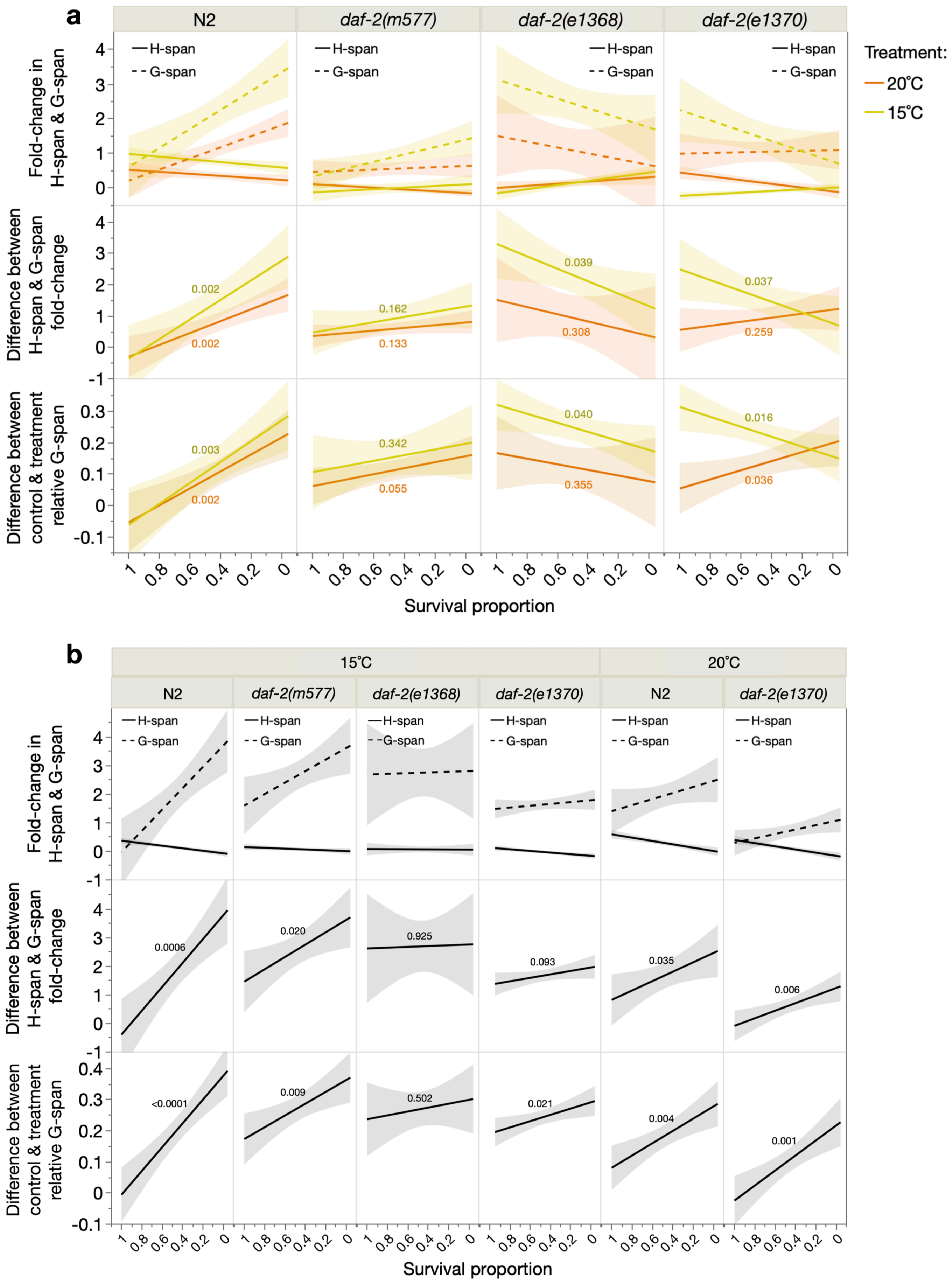
a
N2
daf-2(m577)
daf-2(e1368)
daf-2(e1370)
H-span
G-span
Treatment:
20˚C
15˚C
Fold-change in H-span & G-span
Difference between H-span & G-span fold-change
Difference between control & treatment relative G-span
0.002
0.002
0.162
0.133
0.039
0.308
0.037
0.259
0.003
0.002
0.342
0.055
0.040
0.355
0.016
0.036
Survival proportion
b
15˚C
20˚C
0.0006
0.020
0.925
0.093
0.035
0.006
<0.0001
0.009
0.502
0.021
0.004
0.001
Survival proportion

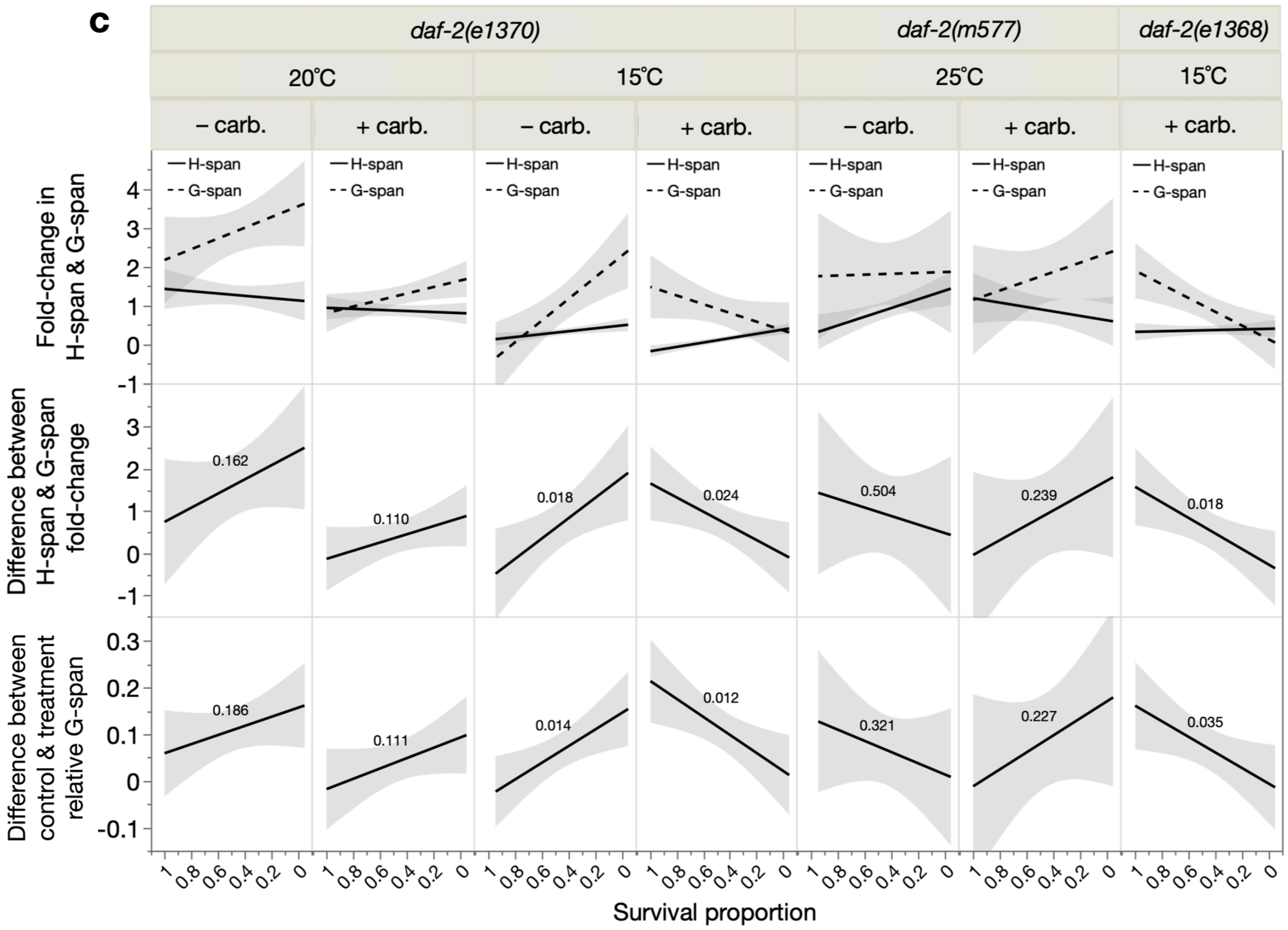

Supplementary Fig. 4 | Extended twilight longevity (ETL) increases gerospan in longer-lived individuals. All longevity treatments causing simultaneous β decrease and G-span^{rel} increase are presented, from three treatment categories: **a**, low temperature, **b**, carbenicillin and **c**, *daf-2(rf)*. N2: wild-type. The **top row of panels** plots the fold-change of absolute H-span and G-span (in response to treatment) over survival proportion (i.e. x-axis left: shorter-lived individuals, x-axis right: longer-lived individuals); in ETL, G-span fold-change is greater than H-span fold-change at lower survival proportions (longer-lived individuals). This difference between G-span and H-span fold-change across survival proportions is plotted and evaluated in the **middle row of panels**; in ETL, this yields a positive relationship, whereas inverted ETL yields a negative relationship. The **bottom row of panels** plots and evaluates the difference between control and treatment G-span^{rel} (from Fig. 2b–g, lower panels) over survival proportion; in ETL, this yields a positive relationship, whereas inverted ETL yields a negative relationship. All panels show a least-squares linear regression with shaded 95% confidence regions, and associated F-test p-values overlaid for middle and bottom panels. We note these regressions may be limited in power by small sample size ($8 \leq n \leq 20$), due to binning and averaging of individual survival proportions (0.05 survival proportion bins) and selective inclusion of only those bins represented in both control and treatment cohorts. However, regardless of statistical significance, all relationship directions (positive or negative) agree with those in Fig. 2b–g, which perform regressions using all (unbinned) uncensored individuals ($104 \leq n \leq 173$).

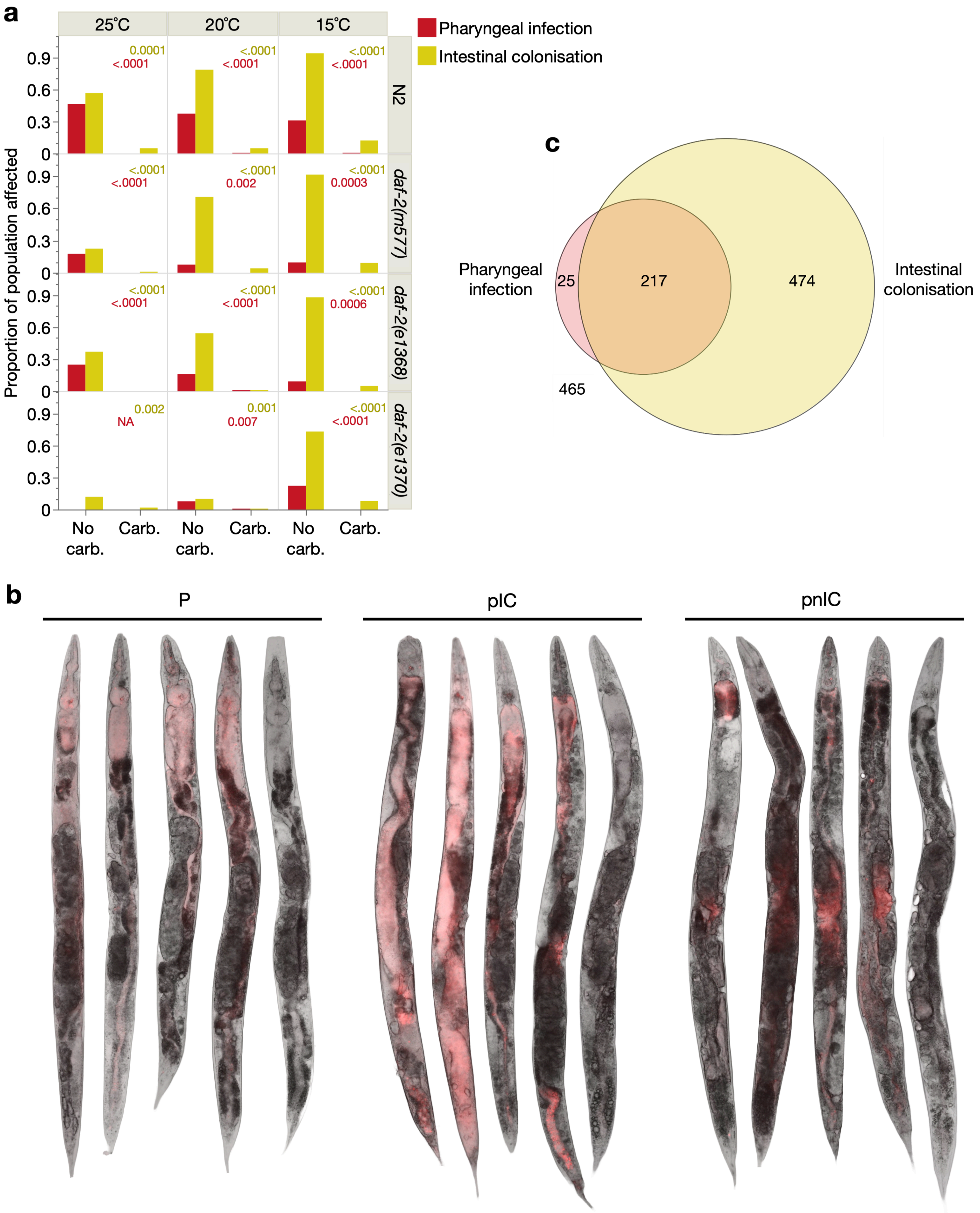

Supplementary Fig. 5 | Pharyngeal and intestinal tissues are colonised by dietary *E. coli* during ageing. **a**, Antibiotic (carbenicillin) treatment abolishes pharyngeal and intestinal colonisation, as scored visually under a Leica MZ8 stereomicroscope (50x total magnification), based on tissue colour, texture and morphology, as described in the methods and performed previously[2]. 95% confidence intervals are shown. Differences in proportion of the population affected were assessed by Pearson's chi-squared test (*p* values

annotated). This was performed on the pool of 3–4 biological replicates (Trials 4–6 for N2 cohorts and Trials 3–6 for all other cohorts, see Supplementary Table 1 for sample sizes), with censored individuals excluded. **b**, Representative images of the range of corpse presentations for each subpopulation (P, pIC, pnIC), taken from a single population (n=45) of nematodes fed throughout life and colonised by RFP-expressing *E. coli* (first four examples in each subpopulation). The fifth (last) example in each subpopulation is of individuals fed standard *E. coli* (lacking RFP expression) throughout life, showing that RFP signal is attributable to bacteria rather than nematode autofluorescence, and that presentation of the described pathologies does not differ between standard and RFP-expressing *E. coli* diets. From left to right, lifespan in days for these specific individuals: P (12, 14, 16, 20, 16), pIC (12, 14, 20, 22, 26), pnIC (16, 20, 24, 34, 30). See Methods for detailed microscopy methodology. Interestingly, epifluorescence microscopy of RFP-expressing *E. coli* reveals its uterine and/or tumoural colonisation in pnIC individuals, which is less readily visible under a dissecting stereomicroscope than pharyngeal and intestinal colonisation. **c**, Venn diagram showing that most P individuals simultaneously have intestinal colonisation by bacteria.

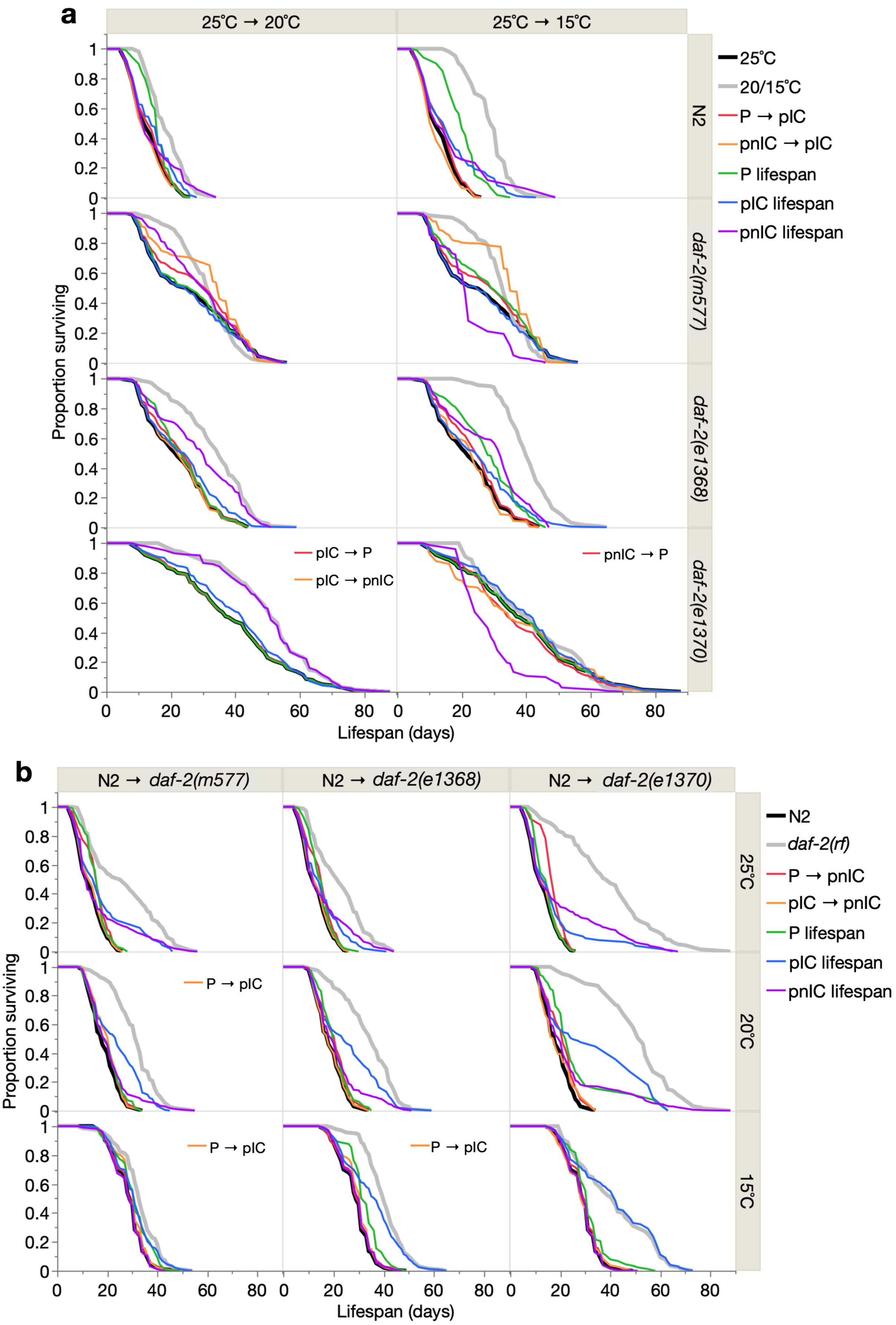

Supplementary Fig. 6 | Effect of life-extending treatments on subpopulation prevalence and lifespan. Effect of **a**, low temperature and **b**, *daf-2(rf)* treatments. Black and grey cohorts are control and treatment cohorts, respectively. Red and orange cohorts are the component effects of changing control subpopulation prevalence to that of the treatment cohort, based on principles of parsimony (see Figure 4e–f), while retaining

control subpopulation lifespans. Green, blue and purple cohorts are the effects of changing control subpopulation lifespan to that of the treatment cohort, while retaining control subpopulation prevalence, for P, pIC and pnIC, respectively. Survival proportions were obtained from Kaplan-Meier survival analysis using mortality pseudofrequencies (in place of frequency) to account for censors (see Methods). Sub-legends reflect different subpopulation prevalence changes for those specific treatments, similarly based on principles of parsimony.

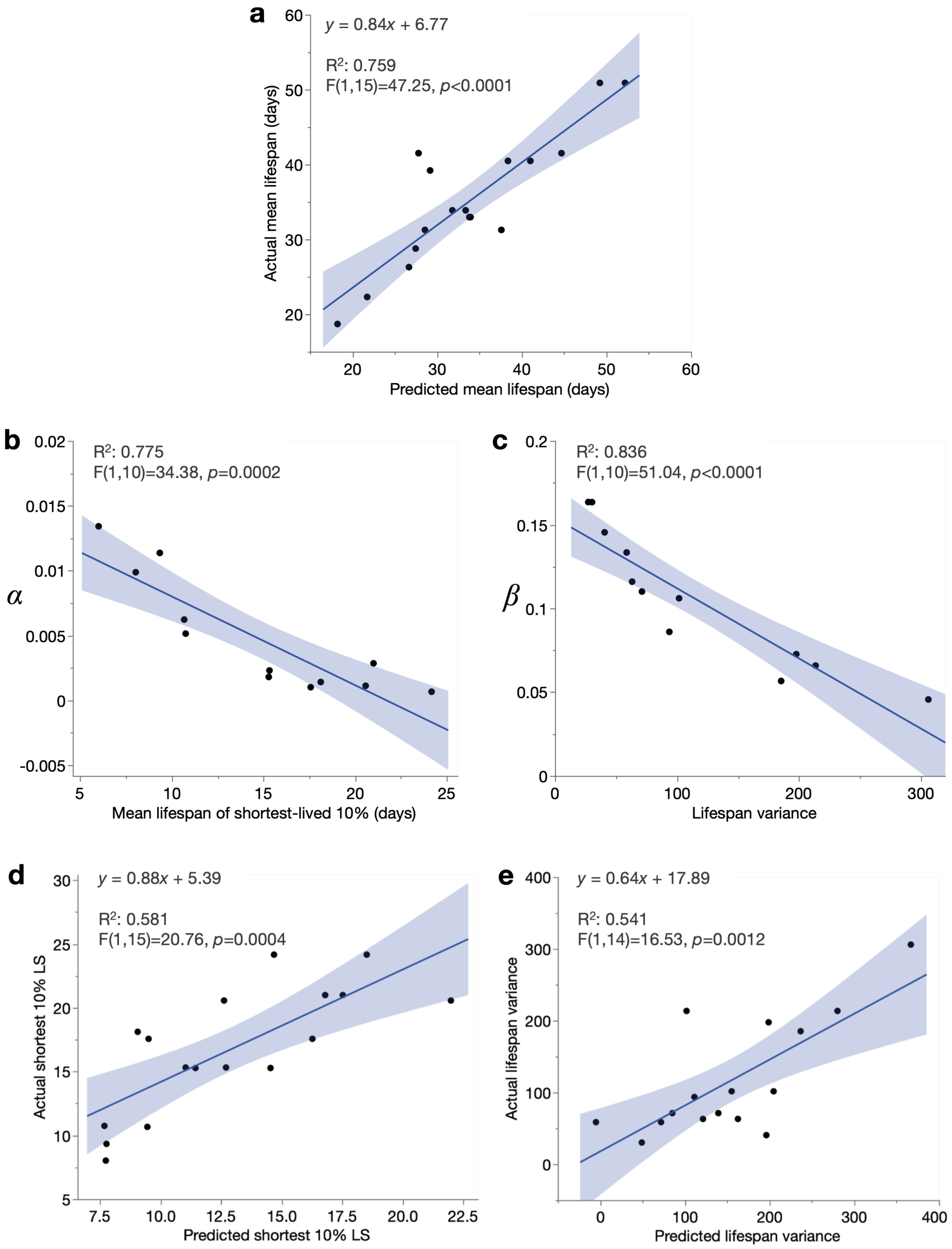

Supplementary Fig. 7 | Methodological background of determining relative contributions to the Gompertz parameters of subpopulation changes. a, Mean lifespan in the 17 treatments (low temperature and *daf-2(rf)*; non-antibiotic background) is strongly predicted by the sum of simulated component changes (in subpopulation prevalence and lifespan) (see Methods for analysis details). **b–c**, Mean lifespan of 10% shortest-lived individuals and lifespan variance are strong respective predictors of α and β in the 12 non-antibiotic cohorts under analysis. **d–e**, Simulated component changes (in subpopulation prevalence and lifespan) predict true changes in mean lifespan of the 10% shortest-lived individuals, and lifespan variance, in the 17 treatments (low temperature and *daf-2(rf)*; non-antibiotic background). In **e**, one outlier treatment (*daf-*

2(e1370) at 20˚C) was excluded, with datapoints x=754, y=198 (Robust Fit Outliers analysis in JMP Pro, Huber method, K=4).

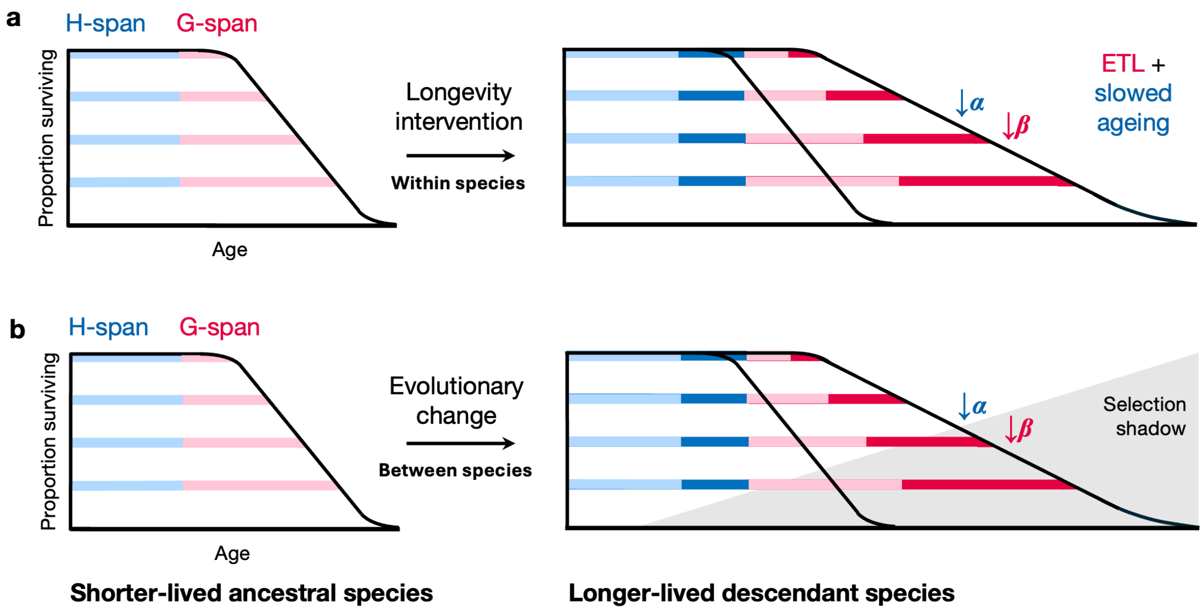

Supplementary Fig. 8 | Potential role for ETL and healthspan extension in the evolution of longevity. Schematic of the biological basis of the Gompertz parameters within and hypothetically, between, species. **a**, Reduction of α by healthspan (H-span) extension and reduction of β by extended twilight longevity (ETL). Each panel depicts variation in health and lifespan in 4 representative individuals; end of bar: point of death, as bordered by the survival curve. Here, as demonstrated in *C. elegans*, the increased variance in lifespan that reduces β arises from inter-individually variable expansion of gerospan (G-span), as part of the phenotypic variability that emerges and progressively increases during ageing. This simplified representation shows no inter-individual variation or change in healthspan (H-span); more accurately, H-span also varies between individuals and increases with lifespan, but to a lesser magnitude than G-span[3]. **b**, Reduction in α and β during evolution of increased longevity (hypothetical scheme). Here again, reduced β reflects increased lifespan variance due to inter-individually variable expansion of G-span, arising from the selection shadow (reduced force of selection) in later life on the evolved genome. This evolutionary ETL is coupled to a less variable expansion of H-span, corresponding to slowed ageing, that reduces α. For simplicity, again no inter-individual variation in H-span is depicted. In neither **a** nor **b** is β a direct metric of biological ageing rate; we suggest it is instead α that is.

			Effect of reducing temperature		Effect of carbenicillin		Effect of *daf-2(rf)*	
Cohort	No. dead/ censors	Mean lifespan (days since L4)	% change vs. 25˚C	*p* vs. 25˚C (Log-Rank)	% change vs. no carb.	*p* vs. no carb. (Log-Rank)	% change vs. N2	*p* vs. N2 (Log-Rank)
N2 25˚C	**[C] 340/47**	**13.9**						
	[1] 95/10	16.4						
	[2] 76/29	13.8						
	[3] 70/1	11.3						
	[4] 33/2	13.7						
	[5] 31/4	11.6						
	[6] 35/1	14.9						
N2 20˚C	**[C] 358/29**	**19.3**	**38.9**	**<0.0001**				
	[1] 96/9	19.7	19.7	0.0001				
	[2] 93/12	19.3	39.8	<0.0001				
	[3] 67/2	19.9	76.1	<0.0001				
	[4] 33/3	20.6	50.5	<0.0001				
	[5] 33/3	18.1	55.7	<0.0001				
	[6] 36/0	17.4	16.9	0.0663				
N2 15˚C	**[C] 328/59**	**28.1**	**102.3**	**<0.0001**				
	[1] 83/22	28.8	75.5	<0.0001				
	[2] 86/19	27.3	98.4	<0.0001				
	[3] 62/8	27.4	142.7	<0.0001				
	[4] 33/3	33.5	145.4	<0.0001				
	[5] 30/5	25.2	116.0	<0.0001				
	[6] 34/2	27.3	83.0	<0.0001				
N2 25˚C carb.	**[C] 341/48**	**19.2**			**38.0**	**<0.0001**		
	[1] 85/20	19.2			16.8	0.0025		
	[2] 83/22	18.0			30.3	<0.0001		
	[3] 70/2	18.2			61.9	<0.0001		
	[4] 34/1	20.8			52.4	<0.0001		
	[5] 35/1	24.0			106.1	<0.0001		
	[6] 34/2	17.6			18.1	0.0235		
N2 20˚C carb.	**[C] 350/39**	**32.6**	**69.8**	**<0.0001**	**68.7**	**<0.0001**		
	[1] 85/20	32.7	70.3	<0.0001	66.2	<0.0001		
	[2] 92/13	34.0	89.6	<0.0001	76.7	<0.0001		
	[3] 70/1	29.0	59.1	<0.0001	46.2	<0.0001		
	[4] 33/3	32.2	54.4	<0.0001	56.5	<0.0001		
	[5] 35/1	32.4	34.9	<0.0001	78.5	<0.0001		
	[6] 35/1	36.4	106.8	<0.0001	108.8	<0.0001		
N2 15˚C carb.	**[C] 337/44**	**43.5**	**126.4**	**<0.0001**	**54.5**	**<0.0001**		
	[1] 81/24	42.0	118.9	<0.0001	45.7	<0.0001		
	[2] 95/10	43.3	141.0	<0.0001	58.2	<0.0001		
	[3] 62/1	38.2	109.3	<0.0001	39.6	<0.0001		
	[4] 32/4	50.2	141.2	<0.0001	49.8	<0.0001		
	[5] 34/2	47.0	95.8	<0.0001	86.8	<0.0001		
	[6] 33/3	47.3	168.7	<0.0001	73.4	<0.0001		
daf-2(m577) 25˚C	**[C] 248/100**	**25.8**					**85.4**	**<0.0001**
	[1] 71/34	27.8					68.9	<0.0001
	[2] 52/53	21.9					58.7	<0.0001
	[3] 21/0	26.6					N/A	N/A
	[4] 32/2	34.7					153.9	<0.0001
	[5] 31/5	26.8					129.9	<0.0001
	[6] 41/6	19.1					27.7	0.0358
daf-2(m577) 20˚C	**[C] 253/98**	**27.7**	**7.4**	**0.164***			**43.3**	**<0.0001**
	[1] 66/39	24.7	-10.8	0.002			25.8	<0.0001
	[2] 68/37	24.0	9.7	0.6874			24.6	<0.0001
	[3] 32/4	36.0	35.3	<0.0001			N/A	N/A
	[4] 26/7	33.2	-4.4	0.057			61.3	<0.0001
	[5] 29/7	28.3	5.8	0.1731			56.2	<0.0001
	[6] 32/4	27.7	45.2	0.0126			58.6	<0.0001
daf-2(m577) 15˚C	**[C] 316/35**	**31.4**	**21.6**	**0.9435***			**11.4**	**<0.0001**
	[1] 84/21	30.0	7.9	0.0411			3.9	0.0699
	[2] 96/9	30.3	38.4	0.1722			10.8	0.0008
	[3] 35/1	34.2	28.5	0.0022			N/A	N/A
	[4] 30/3	33.4	-3.7	0.0205			-0.4	0.1267
	[5] 35/1	32.7	22.1	0.9711			29.9	<0.0001
	[6] 36/0	31.8	67.0	0.0002			16.6	0.0045
daf-2(m577) 25˚C carb.	**[C] 238/88**	**45.0**			**74.7**	**<0.0001**	**134.6**	**<0.0001**
	[1] 78/27	41.8			50.6	<0.0001	117.8	<0.0001
	[2] 56/49	52.5			139.9	<0.0001	192.3	<0.0001
	[3] 18/1	35.8			34.4	0.0005	N/A	N/A
	[4] 29/2	45.8			31.9	0.0032	119.7	<0.0001
	[5] 22/8	41.9			56.4	<0.0001	74.4	<0.0001
	[6] 35/1	45.6			139.3	<0.0001	158.8	<0.0001
daf-2(m577) 20˚C carb.	**[C] 232/103**	**46.4**	**3.1**	**0.1588**	**67.7**	**<0.0001**	**42.5**	**<0.0001**
	[1] 63/42	41.1	-1.8	0.0145	65.9	<0.0001	25.6	<0.0001
	[2] 46/44	45.9	-12.5	<0.0001	91.3	<0.0001	34.9	<0.0001
	[3] 30/2	47.5	32.7	<0.0001	31.8	<0.0001	N/A	N/A
	[4] 27/9	53.7	17.4	<0.0001	62.1	<0.0001	67.1	<0.0001
	[5] 33/3	46.4	10.7	0.0005	63.6	<0.0001	43.1	<0.0001
	[6] 33/3	50.3	10.3	0.0003	81.8	<0.0001	38.1	<0.0001
daf-2(m577) 15˚C carb.	**[C] 308/46**	**53.8**	**19.5**	**<0.0001**	**71.6**	**<0.0001**	**23.8**	**<0.0001**
	[1] 83/22	50.4	20.5	<0.0001	68.1	<0.0001	19.9	<0.0001
	[2] 96/9	53.7	2.3	0.0041	77.2	<0.0001	24.0	<0.0001
	[3] 32/4	51.7	44.6	<0.0001	51.2	<0.0001	N/A	N/A
	[4] 30/6	56.9	24.4	0.0033	70.4	<0.0001	13.3	0.0029
	[5] 34/2	63.6	52.0	<0.0001	94.7	<0.0001	35.4	<0.0001
	[6] 33/3	52.8	15.9	<0.0001	66.0	<0.0001	11.6	0.0098

Cohort	No. dead/ censors	Mean lifespan (days since L4)	Effect of reducing temperature: % change vs. 25˚C	Effect of reducing temperature: *p* vs. 25˚C (Log-Rank)	Effect of carbenicillin: % change vs. no carb.	Effect of carbenicillin: *p* vs. no carb. (Log-Rank)	Effect of *daf-2(rf)*: % change vs. N2	Effect of *daf-2(rf)*: *p* vs. N2 (Log-Rank)
daf-2(e1368) 25˚C	**[C] 326/70**	**22.0**					**58.2**	**<0.0001**
	[1] 100/20	19.6					N/A	N/A
	[2] 85/35	24.2					75.4	<0.0001
	[3] 35/1	23.2					N/A	N/A
	[4] 34/2	23.3					70.6	<0.0001
	[5] 30/6	22.8					95.3	<0.0001
	[6] 42/6	20.7					38.8	0.0006
daf-2(e1368) 20˚C	**[C] 273/110**	**32.4**	**47.4**	**<0.0001**			**67.9**	**<0.0001**
	[1] 78/42	30.7	56.3	<0.0001			N/A	N/A
	[2] 77/44	31.9	32.0	0.0003			65.6	<0.0001
	[3] 31/5	34.6	49.3	<0.0001			N/A	N/A
	[4] 26/8	37.5	61.1	<0.0001			82.7	<0.0001
	[5] 32/4	30.8	35.3	0.0666			69.7	<0.0001
	[6] 29/7	33.2	60.4	<0.0001			90.4	<0.0001
daf-2(e1368) 15˚C	**[C] 345/39**	**40.1**	**82.4**	**<0.0001**			**42.6**	**<0.0001**
	[1] 105/15	41.4	110.8	<0.0001			N/A	N/A
	[2] 108/12	38.5	59.2	<0.0001			40.8	<0.0001
	[3] 34/2	39.3	69.6	<0.0001			N/A	N/A
	[4] 33/3	41.0	75.7	<0.0001			22.2	<0.0001
	[5] 34/2	41.5	82.3	<0.0001			64.9	<0.0001
	[6] 31/5	40.1	93.9	<0.0001			47.0	<0.0001
daf-2(e1368) 25˚C carb.	**[C] 336/48**	**38.1**			**73.3**	**<0.0001**	**98.6**	**<0.0001**
	[1] 108/12	36.8			87.3	<0.0001	N/A	N/A
	[2] 101/19	39.2			62.2	<0.0001	118.5	<0.0001
	[3] 33/3	35.6			53.7	<0.0001	N/A	N/A
	[4] 32/4	37.1			59.1	<0.0001	78.1	<0.0001
	[5] 34/2	40.4			77.6	<0.0001	68.3	<0.0001
	[6] 28/8	40.2			94.3	<0.0001	128.3	<0.0001
daf-2(e1368) 20˚C carb.	**[C] 263/114**	**49.3**	**29.2**	**<0.0001**	**51.9**	**<0.0001**	**51.1**	**<0.0001**
	[1] 67/54	45.5	23.7	<0.0001	48.2	<0.0001	N/A	N/A
	[2] 92/28	47.9	22.0	<0.0001	50.0	<0.0001	40.6	<0.0001
	[3] 25/5	52.3	46.8	<0.0001	51.2	<0.0001	N/A	N/A
	[4] 19/15	56.3	51.7	<0.0001	49.8	<0.0001	74.9	<0.0001
	[5] 29/7	50.6	25.1	<0.0001	64.2	<0.0001	56.1	<0.0001
	[6] 31/5	53.1	32.1	<0.0001	60.0	<0.0001	45.8	<0.0001
daf-2(e1368) 15˚C carb.	**[C] 342/42**	**67.9**	**78.0**	**<0.0001**	**69.1**	**<0.0001**	**56.2**	**<0.0001**
	[1] 108/12	69.1	88.0	<0.0001	67.0	<0.0001	N/A	N/A
	[2] 111/9	67.3	71.5	<0.0001	74.8	<0.0001	55.5	<0.0001
	[3] 30/6	60.6	70.0	<0.0001	54.1	<0.0001	N/A	N/A
	[4] 30/6	66.8	80.0	<0.0001	63.0	<0.0001	33.0	<0.0001
	[5] 35/1	70.3	73.9	<0.0001	69.3	<0.0001	49.5	<0.0001
	[6] 28/8	71.1	76.8	<0.0001	77.1	<0.0001	50.2	<0.0001
daf-2(e1370) 25˚C	**[C] 241/123**	**38.7**					**178.6**	**<0.0001**
	[1] 45/61	38.0					N/A	N/A
	[2] 53/52	37.9					174.7	<0.0001
	[3] 36/0	32.2					N/A	N/A
	[4] 31/2	48.6					255.8	<0.0001
	[5] 34/2	29.3					151.9	<0.0001
	[6] 42/6	46.3					210.2	<0.0001
daf-2(e1370) 20˚C	**[C] 304/52**	**44.4**	**14.7**	**0.0017**			**130.0**	**<0.0001**
	[1] 90/19	39.3	3.4	0.9323			N/A	N/A
	[2] 84/21	39.9	5.3	0.8815			107.0	<0.0001
	[3] 33/1	55.8	73.4	<0.0001			N/A	N/A
	[4] 30/6	58.8	21.0	0.0269			186.0	<0.0001
	[5] 31/5	41.6	41.7	0.0037			129.3	<0.0001
	[6] 36/0	47.8	3.3	0.7321			174.0	<0.0001
daf-2(e1370) 15˚C	**[C] 310/48**	**40.6**	**4.8**	**0.8437**			**44.4**	**<0.0001**
	[1] 94/13	40.1	5.5	0.6179			N/A	N/A
	[2] 90/17	40.1	5.8	0.5505			46.6	<0.0001
	[3] 30/6	44.3	37.8	0.0002			N/A	N/A
	[4] 34/2	34.9	-28.1	0.0051			4.2	0.6074
	[5] 33/3	43.1	46.9	0.0002			71.4	<0.0001
	[6] 29/7	44.2	-4.4	0.2229			62.0	<0.0001
daf-2(e1370) 25˚C carb.	**[C] 257/101**	**55.2**			**42.3**	**<0.0001**	**187.3**	**<0.0001**
	[1] 75/30	63.1			66.0	<0.0001	N/A	N/A
	[2] 70/35	61.2			61.5	<0.0001	240.7	<0.0001
	[3] 32/4	31.1			-3.3	0.5667	N/A	N/A
	[4] 22/8	53.3			9.7	0.4664	156.1	<0.0001
	[5] 30/6	48.0			63.6	<0.0001	99.9	<0.0001
	[6] 28/18	52.9			14.3	0.6046	200.3	<0.0001
daf-2(e1370) 20˚C carb.	**[C] 291/65**	**59.1**	**7.1**	**<0.0001**	**32.9**	**<0.0001**	**81.2**	**<0.0001**
	[1] 87/21	56.1	-11.0	0.5666	42.8	<0.0001	N/A	N/A
	[2] 78/27	53.1	-13.2	0.4668	33.1	<0.0001	55.9	<0.0001
	[3] 33/3	62.7	101.4	<0.0001	12.3	0.0095	N/A	N/A
	[4] 28/7	76.1	42.6	<0.0001	29.4	<0.0001	136.5	<0.0001
	[5] 33/3	58.1	21.1	0.0322	39.7	0.0001	79.4	<0.0001
	[6] 32/4	61.5	16.2	0.0043	28.6	0.0006	68.7	<0.0001
daf-2(e1370) 15˚C carb.	**[C] 299/46**	**65.8**	**19.3**	**<0.0001**	**61.9**	**<0.0001**	**51.3**	**<0.0001**
	[1] 86/21	70.3	11.4	<0.0001	75.3	<0.0001	N/A	N/A
	[2] 89/16	65.8	7.6	0.0002	64.2	<0.0001	52.1	<0.0001
	[3] 20/5	51.6	65.9	<0.0001	16.4	0.0785	N/A	N/A
	[4] 34/2	53.3	-0.1	0.8327	52.5	0.0003	6.1	0.4075
	[5] 34/2	73.4	52.8	<0.0001	70.1	<0.0001	56.1	<0.0001
	[6] 36/0	67.4	27.4	<0.0001	52.4	<0.0001	42.3	<0.0001

Supplementary Table 1 | Lifespan data for all cohorts and trials, and effect on lifespan of reducing temperature, antibiotic treatment and *daf-2(rf)*. N2, wild-type. [C], combined (pooled) data from all trials; [*n*], trial number; carb., carbenicillin. *, cohorts where Log-Rank $p>0.05$, due to crossing survival curves and greater changes in early than late mortality; the Wilcoxon test is more appropriate to detect lifespan differences in these conditions ($p<0.0015$; data not shown). The 6 trials were performed sequentially over 34 months. In Trials 1–3, experiments for the 24 cohorts were not always performed together due to practical constraints. In this table, intra-trial comparisons between such cohorts were therefore withheld, and these exclusions (affecting a small proportion of within-trial comparisons: 30/276) are labelled (N/A). However, most analyses performed in this study utilise pooled data (Trials 3–6) from multiple trials (see Methods); in these analyses, the above-excluded comparisons were included, given that pooled analyses by nature compare different trials, and additionally dilute inter-trial noise.

Cohort	Kolmogorov-Smirnov test				Anderson-Darling test			
	Trials 1–6		Trials 3–6		Trials 1–6		Trials 3–6	
	Test statistic	*p* value	Test statistic	*p* value	Test statistic	*p* value	Test statistic	*p* value
N2 25˚C	0.0990	0.0010	0.1331	0.0030	0.4325	0.8260	0.5875	0.6841
N2 20˚C	0.0783	0.0144	0.0886	0.0952	0.5400	0.7153	0.4964	0.7648
N2 15˚C	0.0783	0.0134	0.0941	0.0714	0.5501	0.7081	4.3959	0.0088
N2 25˚C carb.	0.0894	0.0028	0.0802	0.1628	0.4875	0.7702	0.4016	0.8602
N2 20˚C carb.	0.0375	0.5813	0.0327	0.9682	0.4407	0.8136	0.4455	0.8220
N2 15˚C carb.	0.0415	0.4725	0.0575	0.5521	2.3866	0.0618	0.5353	0.7465
daf-2(m577) 25˚C	0.1443	0.0002	0.1294	0.0146	1.3159	0.2418	0.9701	0.4109
daf-2(m577) 20˚C	0.0829	0.0142	0.1060	0.0644	0.5939	0.6713	0.4964	0.7694
daf-2(m577) 15˚C	0.1236	0.0002	0.0831	0.2358	1.1436	0.2919	5.0822	0.0076
daf-2(m577) 25˚C carb.	0.0701	0.0672	0.0682	0.5457	0.7175	0.5651	0.2733	0.9574
daf-2(m577) 20˚C carb.	0.0667	0.0810	0.0887	0.1732	0.1615	0.9974	41.6069	0.0002
daf-2(m577) 15˚C carb.	0.0410	0.5305	0.0643	0.5041	0.2918	0.9466	0.4773	0.7882
daf-2(e1368) 25˚C	0.1058	0.0006	0.1031	0.0548	0.7825	0.5253	0.6964	0.5905
daf-2(e1368) 20˚C	0.0471	0.3285	0.0521	0.7469	1.0214	0.3699	0.1748	0.9970
daf-2(e1368) 15˚C	0.1486	0.0002	0.1161	0.0322	4.2711	0.0054	0.9692	0.4117
daf-2(e1368) 25˚C carb.	0.1370	0.0002	0.1247	0.0160	2.4469	0.0600	0.9452	0.4199
daf-2(e1368) 20˚C carb.	0.0584	0.1262	0.0703	0.4355	4.0242	0.0086	0.2774	0.9524
daf-2(e1368) 15˚C carb.	0.0924	0.0028	0.0741	0.3441	0.6852	0.5879	0.2630	0.9678
daf-2(e1370) 25˚C	0.0554	0.1908	0.0573	0.5931	0.3347	0.9200	0.2427	0.9710
daf-2(e1370) 20˚C	0.0765	0.0270	0.0912	0.1454	1.5729	0.1792	0.2636	0.9660
daf-2(e1370) 15˚C	0.0907	0.0040	0.0948	0.1212	1.2348	0.2567	0.8100	0.4907
daf-2(e1370) 25˚C carb.	0.0484	0.3503	0.1011	0.0752	0.4349	0.8324	0.6524	0.6291
daf-2(e1370) 20˚C carb.	0.0793	0.0208	0.1119	0.0386	1.4133	0.2178	1.0364	0.3643
daf-2(e1370) 15˚C carb.	0.0694	0.0588	0.1130	0.0498	0.6600	0.6087	1.0899	0.3403

Supplementary Table 2 | Additional goodness-of-fit test statistics for all cohorts. Test statistics and *p* values for parametric bootstrap Kolmogorov-Smirnov and Anderson-Darling tests, to assess goodness-of-fit of the 24 cohorts to Gompertz distributions. The K-S test assesses the region of worst fit (here as the maximum absolute deviation between the empirical and Gompertzian cumulative distribution functions), while the A-D test assesses overall, tail-weighted fit (here as the sum of squared, tail-weighted deviations between these functions across all ages). For each cohort, observed K-S and A-D statistics were compared against null distributions generated from 5000 parametric bootstrap populations simulated in JMP under the fitted Gompertz distribution of interest (MLE parameters obtained in WinModest), by generating random survival proportions (assuming a Uniform(0,1) distribution) and population sizes matching the observed populations (including censored individuals). Monte Carlo *p* values were computed as the proportion of bootstrap K-S/A-D statistics greater than or equal to the observed statistic, applying the Phipson–Smyth correction (addition of 1 to numerator and denominator). These tests were performed for the pool of all trials (1–6) as well as the pool of only those trials in which locomotory health was scored (3–6). *p* values less than 0.05 are shaded in grey.

a

Effect of low temperature on β			Control (25˚C + carbenicillin)							
			N2		*daf-2(m577)*		*daf-2(e1368)*		*daf-2(e1370)*	
			H-span	G-span	H-span	G-span	H-span	G-span	H-span	G-span
Treatment (20 or 15˚C + carbenicillin)	20˚C	Abs.	-0.06	-0.24						
		Rel.	-19.7	-62.1						
	15˚C	Abs.	-0.14	-0.30						
		Rel.	-47.0	-78.5						
	20˚C	Abs.			0.02	0.01				
		Rel.			23.1	14.5				
	15˚C	Abs.			-0.03	-0.01				
		Rel.			-28.3	-6.6				
	20˚C	Abs.					-0.12	0.01		
		Rel.					-38.3	9.2		
	15˚C	Abs.					-0.19	-0.06		
		Rel.					-63.7	-45.0		
	20˚C	Abs.							0.048	-0.005
		Rel.							67.3	-12.4
	15˚C	Abs.							0.001	0.016
		Rel.							0.8	44.0

b

Effect of carbenicillin on β		Control (– carbenicillin)											
		N2				*daf-2(m577)*		*daf-2(e1368)*		*daf-2(e1370)*			
		20˚C		15˚C		15˚C		15˚C		20˚C		15˚C	
		H-span	G-span	H-span	G-span	H-span	G-span	H-span	G-span	H-span	G-span	H-span	G-span
Treatment (+ carbenicillin)	Abs.	0.06	-0.13										
	Rel.	33.2	-46.5										
	Abs.			0.01	-0.15								
	Rel.			10.6	-63.9								
	Abs.					-0.058	-0.063						
	Rel.					-43.7	-43.3						
	Abs.							-0.028	-0.005				
	Rel.							-19.9	-6.5				
	Abs.									0.028	-0.029		
	Rel.									31.6	-47.5		
	Abs.											-0.01	-0.02
	Rel.											-7.5	-27.7

c

Effect of *daf-2(rf)* on β			Control (N2)											
			– carbenicillin						+ carbenicillin					
			25˚C		20˚C		15˚C		25˚C		20˚C		15˚C	
			H-span	G-span	H-span	G-span	H-span	G-span	H-span	G-span	H-span	G-span	H-span	G-span
Treatment (*daf-2* mutation)	*daf-2(m577)*	Abs.	-0.17	-0.08					-0.18	-0.30				
		Rel.	-71.2	-47.5					-64.1	-77.1				
	daf-2(e1368)	Abs.											-0.04	-0.01
		Rel.											-27.6	-17.7
	daf-2(e1370)	Abs.			-0.08	-0.21								
		Rel.			-48.3	-77.5								
		Abs.					-0.06	-0.16						
		Rel.					-44.3	-68.0						
		Abs.									-0.113	-0.115		
		Rel.									-48.9	-77.9		
		Abs.											-0.08	-0.03
		Rel.											-53.4	-35.9

Supplementary Table 3 | Effect of changes in H-span and G-span on the β parameter. All longevity treatments causing simultaneous β decrease and G-span^{rel} increase are presented, from three treatment categories: **a**, low temperature, **b**, carbenicillin, and **c**, *daf-2(rf)*. N2, wild-type. Shaded grey cells indicate treatments causing extended twilight longevity (ETL), and unshaded cells indicate treatments causing inverted ETL (see main text). Treatments where G-span causes greater reduction in β than H-span are enclosed by a bold border. Both absolute (Abs.) and relative percentage (Rel.) effects are shown, with the sign (positive/negative) indicating direction of change. For this analysis, β was obtained by maximum likelihood estimation in WinModest using H-span and G-span data (instead of lifespan data), for control and treatment cohorts, and the change (between control and treatment) quantified to estimate the separate contributions of H-span and G-span to lifespan β (H-span and G-span sum to lifespan and have approximately additive effects on β).

a

Effect of low temperature on lifespan standard deviation			Control (25˚C + carbenicillin)							
			N2		*daf-2(m577)*		*daf-2(e1368)*		*daf-2(e1370)*	
			H-span	G-span	H-span	G-span	H-span	G-span	H-span	G-span
Treatment (20 or 15˚C + carbenicillin)	20˚C	Abs.	0.8	3.7						
		Rel.	23.6	153.0						
	15˚C	Abs.	2.3	7.8						
		Rel.	69.8	324.1						
	20˚C	Abs.			-1.8	-0.5				
		Rel.			-20.5	-5.9				
	15˚C	Abs.			0.05	3.80				
		Rel.			0.5	44.6				
	20˚C	Abs.					1.8	1.1		
		Rel.					45.2	20.2		
	15˚C	Abs.					4.7	7.5		
		Rel.					120.0	136.7		
	20˚C	Abs.							-3.3	6.1
		Rel.							-29.1	51.3
	15˚C	Abs.							-1.0	3.9
		Rel.							-9.0	32.4

b

Effect of carbenicillin on lifespan standard deviation		Control (– carbenicillin)											
		N2				*daf-2(m577)*		*daf-2(e1368)*		*daf-2(e1370)*			
		20˚C		15˚C		15˚C		15˚C		20˚C		15˚C	
		H-span	G-span	H-span	G-span	H-span	G-span	H-span	G-span	H-span	G-span	H-span	G-span
Treatment (+ carbenicillin)	Abs.	-0.9	3.7										
	Rel.	-19.0	157.0										
	Abs.			-0.5	6.9								
	Rel.			-8.6	208.2								
	Abs.					1.5	8.1						
	Rel.					21.1	188.1						
	Abs.							1.4	6.1				
	Rel.							19.2	87.4				
	Abs.									-2.4	8.1		
	Rel.									-23.5	81.8		
	Abs.											-0.5	8.0
	Rel.											-4.9	104.4

c

Effect of *daf-2(rf)* on lifespan standard deviation		Control (N2)											
		– carbenicillin						+ carbenicillin					
		25˚C		20˚C		15˚C		25˚C		20˚C		15˚C	
		H-span	G-span	H-span	G-span	H-span	G-span	H-span	G-span	H-span	G-span	H-span	G-span
**Treatment (*daf-2* mutation)**													
daf-2(m577)	Abs.	6.4	2.9					5.4	6.1				
	Rel.	180.3	127.9					166.0	256.4				
daf-2(e1368)	Abs.											3.1	2.9
	Rel.											56.4	28.4
daf-2(e1370)	Abs.			5.4	7.5								
	Rel.			108.9	319.5								
	Abs.					4.7	4.4						
	Rel.					77.0	133.6						
	Abs.									3.9	11.9		
	Rel.									97.3	196.7		
	Abs.											4.7	5.6
	Rel.											84.1	54.9

Supplementary Table 4 | Effect of changes in H-span and G-span on lifespan standard deviation. All longevity treatments causing simultaneous β decrease and G-span^{rel} increase are presented, from three treatment categories: **a**, low temperature, **b**, carbenicillin, and **c**, *daf-2(rf)*. N2, wild-type. Shaded grey cells indicate treatments causing extended twilight longevity (ETL), and unshaded cells indicate treatments causing inverted ETL (see main text). Treatments where G-span causes greater increase in lifespan standard deviation than H-span are enclosed by a bold border. Both absolute (Abs.) and relative percentage (Rel.) effects are shown, with the sign (positive/negative) indicating direction of change (positive changes are related to β reduction). For this analysis, standard deviation was obtained for H-span and G-span, for control and treatment cohorts, and the change (between control and treatment) quantified to estimate the separate contributions of H-span and G-span to lifespan standard deviation (H-span and G-span sum to lifespan and have approximately additive effects on lifespan standard deviation).

a

Effect of H-span expansion in low temperature treatment		Control (25˚C)											
		N2				*daf-2(m577)*		*daf-2(e1368)*				*daf-2(e1370)*	
		– carb.		+ carb.		– carb.		– carb.		+ carb.		+ carb.	
		α	β	α	β	α	β	α	β	α	β	α	β
**Treatment (20 or 15˚C)**	20˚C	**-98**	**-33**										
	15˚C	**-250**	**-49**										
	20˚C			**-53**	2								
	15˚C			**-105**	-25								
	20˚C					**-586**	**30**						
	15˚C					**-691**	**39**						
	20˚C							**-198**	**-6**				
	15˚C							**-437**	**42**				
	20˚C									**0.5**	**-525**		
	15˚C									**10**	**-725**		
	20˚C											**-46**	**45**

b

Effect of H-span expansion in carbenicillin treatment	Control (– carbenicillin)													
	N2						*daf-2(m577)*				*daf-2(e1368)*			
	25˚C		20˚C		15˚C		25˚C		20˚C		25˚C		20˚C	
	α	β	α	β	α	β	α	β	α	β	α	β	α	β
**Treatment (+ carbenicillin)**	**-152**	**24**												
			**-95**	**44**										
					**-47**	**36**								
							**-951**	**57**						
									**-62**	**53**				
											**-527**	**93**		
													**-110**	**60**

c

Effect of H-span expansion in *daf-2(rf)* treatment		Control (N2)											
		– carbenicillin						+ carbenicillin					
		25˚C		20˚C		15˚C		25˚C		20˚C		15˚C	
		α	β	α	β	α	β	α	β	α	β	α	β
Treatment (*daf-2(rf)* mutation)	*daf-2(m577)*	**46**	**-185**										
	daf-2(m577)			**-88**	**-51**								
	daf-2(m577)					**-22**	-18						
	daf-2(m577)							**-127**	**-60**				
	daf-2(m577)									**-57**	-28		
	daf-2(m577)											**6**	**-75**
	daf-2(e1368)	**-24**	**-127**										
	daf-2(e1368)			**-86**	**-81**								
	daf-2(e1368)					**-89**	12						
	daf-2(e1368)							**-208**	**80**				
	daf-2(e1368)									**-100**	-29		
	daf-2(e1368)											**-35**	-33
	daf-2(e1370)	**-179**	**-240**										
	daf-2(e1370)			**-177**	**-67**								
	daf-2(e1370)					**7**	**-89**						
	daf-2(e1370)							**-115**	**-157**				
	daf-2(e1370)									**-105**	-44		
	daf-2(e1370)											**20**	**-123**

Supplementary Table 5 | Effect of absolute H-span increase on *α* and *β*. All longevity treatments causing H-span^{abs} increase are presented, from three treatment categories: **a**, low temperature, **b**, carbenicillin, and **c**, *daf-2(rf)*. N2, wild-type. Shaded grey cells indicate treatments causing extended twilight longevity (ETL). Relative percentage effects are shown, with the sign (positive/negative) indicating direction of change, and statistically significant (p<0.05) effects are in bold text. Effects of α and β were estimated as changes in the y-intercept and gradient of the least-squares linear regression model of H-span^{abs} over survival proportion (e.g. as plotted in Fig. 2b–g, upper panels). Increases in y-intercept and gradient correspond, respectively, to decreases in α and β. Statistical significance of these changes was assessed by two-tailed regression t-tests on model parameter estimates (y-intercept, treatment term; gradient, interaction term between treatment and survival proportion).

								Effect of reducing temperature		Effect of *daf-2(rf)*	
Condition	Cohort	No. dead/ censors	Mean lifespan (days since L4)	% change vs. P	*p* vs. P (Log-Rank)	% change vs. pIC	*p* vs. pIC (Log-Rank)	% change vs. 25˚C	*p* vs. 25˚C (Log-Rank)	% change vs. N2	*p* vs. N2 (Log-Rank)
N2 25˚C	P	46	9.5								
	pIC	20	14.7	54.4	<0.0001						
	pnIC	33	17.9	87.6	<0.0001	21.5	0.0770*				
	Combined	99/7	13.4								
N2 20˚C	P	38	14.6					53.4	<0.0001		
	pIC	45	20.8	42.3	<0.0001			41.3	<0.0001		
	pnIC	19	21.6	47.4	<0.0001	3.6	0.1225	20.5	0.0037		
	Combined	102/6	18.7					39.1	<0.0001		
N2 15˚C	P	30	24.2					153.2	<0.0001		
	pIC	61	30.9	27.7	<0.0001			109.5	<0.0001		
	pnIC	6	30.2	24.9	0.0739	-2.3	0.3779	68.5	0.0008		
	Combined	97/10	28.8					114.0	<0.0001		
daf-2(m577) 25˚C	P	22	14.6							52.5	<0.0001
	pIC	10	38.7	166.0	<0.0001					162.7	<0.0001
	pnIC	93	27.6	89.5	<0.0001	-28.8	0.3309*			54.0	<0.0001
	Combined	125/13	26.3							95.6	<0.0001
daf-2(m577) 20˚C	P	9	16.8					15.6	0.3766	15.0	0.2530
	pIC	76	32.1	91.0	<0.0001			-17.0	0.0130	54.3	<0.0001
	pnIC	34	32.8	95.2	<0.0001	2.2	0.1298	19.1	0.3506	52.2	<0.0001
	Combined	119/22	31.3					18.9	0.6186*	67.2	<0.0001
daf-2(m577) 15˚C	P	13	30.9					112.0	<0.0001	27.7	0.0088
	pIC	111	34.4	11.4	0.6031			-11.2	0.1587	11.3	0.0002
	pnIC	12	22.2	-28.2	0.0306	-35.5	<0.0001	-19.6	0.0976	-26.5	0.1409
	Combined	136/5	33.0					25.4	0.3171*	14.6	<0.0001
daf-2(e1368) 25˚C	P	35	13.3							39.9	<0.0001
	pIC	21	25.2	88.6	<0.0001					70.8	<0.0001
	pnIC	85	25.1	88.0	<0.0001	-0.3	0.6036			40.2	<0.0001
	Combined	141/15	22.3							65.9	<0.0001
daf-2(e1368) 20˚C	P	19	19.5					46.3	0.0003	33.4	0.0007
	pIC	45	36.5	87.1	<0.0001			45.1	<0.0001	75.4	<0.0001
	pnIC	54	36.2	85.5	<0.0001	-0.9	0.893	44.3	<0.0001	67.9	<0.0001
	Combined	118/24	33.9					51.9	<0.0001	81.2	<0.0001
daf-2(e1368) 15˚C	P	12	36.0					169.6	<0.0001	48.9	<0.0001
	pIC	104	41.8	16.0	0.0094			65.9	<0.0001	35.3	<0.0001
	pnIC	16	35.5	-1.2	0.8754	-14.9	0.0078	41.6	0.0026	17.8	0.9213
	Combined	132/12	40.5					81.5	<0.0001	40.7	<0.0001
daf-2(e1370) 25˚C	P	0	N/A							N/A	N/A
	pIC	17	35.9	N/A	N/A					143.3	0.0003
	pnIC	126	39.7	N/A	N/A	10.6	0.8136			121.5	<0.0001
	Combined	143/10	39.2							191.6	<0.0001
daf-2(e1370) 20˚C	P	10	36.9					N/A	N/A	151.8	<0.0001
	pIC	5	53.0	43.8	0.5439			47.8	0.5539	154.5	0.0002
	pnIC	115	52.0	41.1	0.0877*	-1.9	0.8634	31.1	0.0001	140.9	<0.0001
	Combined	130/12	50.9					29.8	0.0001	172.2	<0.0001
daf-2(e1370) 15˚C	P	28	32.4					N/A	N/A	34.1	0.0004
	pIC	65	51.5	58.8	<0.0001			43.5	0.1455*	66.7	<0.0001
	pnIC	33	28.2	-13.0	0.1303	-45.2	<0.0001	-28.9	<0.0001	-6.5	0.6324
	Combined	126/18	41.5					5.9	0.9204	44.3	<0.0001

Supplementary Table 6 | Lifespan data for P, pIC and pnIC subpopulations in non-carbenicillin cohorts, their differences and effect on them of low temperature and *daf-2(rf)* treatment. N2, wild-type. Mean lifespan was obtained by Kaplan-Meier analysis using age-specific mortality pseudofrequencies, to allow unbiased incorporation of censors in subpopulation analyses (see Methods). Mean lifespan of the subpopulations and combined population was obtained by Kaplan-Meier analysis using these mortality pseudofrequencies. *, cohorts where Log-Rank $p>0.05$, due to crossing survival curves and/or greater changes in early than late mortality; the Wilcoxon test is more appropriate to detect lifespan differences in these conditions ($0.01<p<0.05$ for inter-subpopulation comparisons, and $0.0002<p<0.006$ for treatment effects; data not shown).

Condition	Cohort	No. dead/ censors	β	% change vs. combined	p vs. combined (LRT)
N2 25˚C	P	46	0.38456	135.2	<0.0001
	pIC	20	0.18510	13.2	0.6039
	pnIC	33	0.27666	69.2	0.0032
	**Combined**	**99/7**	**0.16350**		
N2 20˚C	P	38	0.33946	107.7	<0.0001
	pIC	45	0.26673	63.2	0.0018
	pnIC	19	0.14675	-10.2	0.6207
	**Combined**	**102/6**	**0.16346**		
N2 15˚C	P	30	0.21114	45.2	0.0246
	pIC	61	0.21046	44.7	0.0025
	pnIC	6	0.07728	-46.9	0.0574
	**Combined**	**97/10**	**0.14543**		
daf-2(m577) 25˚C	P	22	0.16444	190.2	0.0028
	pIC	10	0.23111	307.8	0.0004
	pnIC	93	0.05929	4.6	0.8180
	**Combined**	**125/13**	**0.05667**		
daf-2(m577) 20˚C	P	9	0.11551	4.9	0.9034
	pIC	76	0.17480	58.8	0.0003
	pnIC	34	0.08814	-19.9	0.1969
	**Combined**	**119/22**	**0.11009**		
daf-2(m577) 15˚C	P	13	0.11613	-13.0	0.6464
	pIC	111	0.14486	8.6	0.4538
	pnIC	12	0.11676	-12.5	0.8922
	**Combined**	**136/5**	**0.13341**		
daf-2(e1368) 25˚C	P	35	0.14304	66.3	0.0437
	pIC	21	0.13201	53.5	0.1202
	pnIC	85	0.10012	16.4	0.3649
	**Combined**	**141/15**	**0.08599**		
daf-2(e1368) 20˚C	P	19	0.12294	15.9	0.5731
	pIC	45	0.11665	10.0	0.5193
	pnIC	54	0.13545	27.7	0.1064
	**Combined**	**118/24**	**0.10606**		
daf-2(e1368) 15˚C	P	12	0.19005	63.9	0.1030
	pIC	104	0.11830	2.0	0.8546
	pnIC	16	0.15642	34.9	0.2487
	**Combined**	**132/12**	**0.11598**		
daf-2(e1370) 25˚C	P	0	N/A	N/A	N/A
	pIC	17	0.03747	-17.9	0.6227
	pnIC	126	0.04657	2.1	0.8985
	**Combined**	**143/10**	**0.04563**		
daf-2(e1370) 20˚C	P	10	0.04380	-39.7	0.1972
	pIC	5	0.15907	118.9	0.1117
	pnIC	115	0.07471	2.8	0.8123
	**Combined**	**130/12**	**0.07268**		
daf-2(e1370) 15˚C	P	28	0.07444	13.0	0.5900
	pIC	65	0.10575	60.6	0.0015
	pnIC	33	0.09422	43.1	0.0951
	**Combined**	**126/18**	**0.06586**		

Supplementary Table 7 | β parameters for P, pIC and pnIC subpopulations in non-carbenicillin cohorts, and their differences. N2, wild-type. β parameters were obtained by maximum likelihood estimation using mortality pseudofrequencies (in place of standard mortality frequencies) (see Methods). LRT, likelihood ratio test, used here to assess statistical significance of β differences between subpopulations and whole populations (combined).

References

1 Podshivalova, K., Kerr, R. & Kenyon, C. How a mutation that slows aging can also disproportionately extend end-of-life decrepitude. *Cell Reports* **19**, 441–450 (2017).

2 Zhao, Y. *et al.* Two forms of death in aging Caenorhabditis elegans. *Nat. Commun.* **8**, 15458 (2017).

3 Zhang, W. *et al.* Extended twilight among isogenic C. elegans causes a disproportionate scaling between lifespan and health. *Cell Syst.* **3**, 333-345 (2016).
