# Supplementary material for: Slowed Gompertzian ageing in long-lived C. elegans results from expansion of decrepitude, not decelerated ageing
Source: Nat Commun. 2026 Apr 13;17:5182. doi: 10.1038/s41467-026-71780-7 (PMC13254367; doi:10.1038/s41467-026-71780-7)
Supplement: Supplementary file 3 — Transparent Peer Review file [file 41467_2026_71780_MOESM3_ESM.pdf]

nature portfolio

# Peer Review File

# Slowed Gompertzian ageing in long-lived *C. elegans* results from expansion of decrepitude, not decelerated ageing

Corresponding Author: Professor David Gems

**This file contains all reviewer reports in order by version, followed by all author rebuttals in order by version.**

Version 0:

Reviewer comments:

Reviewer #1

(Remarks to the Author)
This study reinterprets Gompertz mortality parameters in C. elegans. Contrary to convention, it argues that reductions in β reflect heterogeneity and extended late-life decrepitude (gerospan), while reductions in α better track healthspan extension and slower biological ageing. Linking longitudinal locomotor health to survival, the authors propose α, not β, captures ageing pace.
There is little doubt that this paper merits publication. In fact, it reads almost as two papers in one: a hierarchy in which one layer generates evidence for reinterpretation of the Gompertz parameters, and another delves deeply into the biology of ageing. The breadth of the experiments and the strength of the data are remarkable. Yet unless a study of this complexity is presented with exceptional clarity, its impact beyond the inner sanctum of cell and molecular biology, reaching, for example, demographers and population biologists, will remain limited. Demographers in particular, are unlikely to fully understand or even engage with the results without clear guidance through the visual and conceptual density.
In addition to the need to make the paper more accessible (outlined later), I have two conceptual concerns that I would like the authors to address more clearly in the manuscript.
1. Justification for temperature as treatment. While reduced temperature reliably extends C. elegans lifespan, in my view its interpretive value for ageing is limited. Lower temperature slows virtually all biological processes including development, reproduction, and decline through thermodynamic effects, akin to redefining a “day” as 12 hours instead of 24. The clock runs more slowly, so individuals live longer in calendar time, but nothing has fundamentally changed about the mechanisms of ageing. By contrast, interventions such as dietary restriction or daf-2 mutations alter how long worms remain healthy relative to their developmental and reproductive schedules. These pathway-level manipulations reflect genuine changes in ageing pace, whereas temperature effects reflect environmental pacing. Thus, cold exposure demonstrates lifespan plasticity but is a weaker test of mechanistic theories of ageing. The authors cite no papers on temperature as a life-extending method that situates it as an slowed aging concept.
2. Frailty versus gerospan. The authors reinterpret β as a marker of heterogeneity in gerospan rather than as the rate of biological aging. Although they cite Vaupel’s 1979 frailty model, they do not engage with its central implication that β still represents the rate of aging within sub-cohorts, with the pooled β emerging from their mixture. By contrast, their gerospan framework discards this interpretation altogether, treating β solely as a metric of variation in decrepitude across individuals. Thus, both models place heterogeneity at the center of demographic aging, but they diverge sharply on whether β should be viewed as a rate of aging or a measure of dispersion. The authors need to make this distinction clear so readers can understand how their reinterpretation departs from established demographic theory.
I see two levels of clarity that need to be improved in the manuscript. The first concerns the overarching hypothesis or framework. With 46 treatments and a bewildering number of “moving parts” (graphs; sub-graphs; sub-sub-graphs), the big experimental picture is extremely difficult to grasp. Readers would benefit from a clear, anchoring framework, something akin to a guiding syllogism (“if premises n are met, then X, Y, Z”), that makes the logical structure of the argument explicit. Such a framework would help orient the reader as they work their way through the results.
The second concerns the details (i.e., Results). The figures in this study demand readers process patterns at several levels: within plots (curvature of survival curves, divergence of lines), between subfigures (temperature vs. antibiotic vs. daf-2 effects), and across figures (gerospan expansion, variability, α vs. β, necropsy subpopulations). They must parse directional shifts (α vs. β), proportional changes in healthspan vs. gerospan, cross-condition contrasts, subpopulation divergences, statistical signals, and conceptual overlays like “extended twilight longevity.” The cumulative expectation is that readers track 46 treatments, distinguish absolute from relative measures, reinterpret α and β, connect demographic curves with individual outcomes, and integrate diverse statistical modes—spread across 5 figures with ~58 distinct plots. In sum, this is a

bewildering interpretive task requiring alignment of scales, metrics, and interventions. For this reason, I strongly recommend that the authors revisit the Results and consider a clearer, more structured way of presenting both the figures and the accompanying narrative, so that readers outside of the immediate field can engage with and appreciate the significance of their findings. I understand this will be no easy task, but without a clearer and more structured presentation, many readers will be left unable to follow the argument and may simply defer to the authors' authority rather than genuinely engaging with the evidence.

Possible syllogism:
Premise 1 (health vs. decrepitude): If locomotor health can be separated into two phases, H-span and G-span, then we can track healthspan and decrepitude separately across lifespan interventions.
Premise 2 (effects of interventions): If interventions such as lower temperature, antibiotics, and daf-2 mutations extend mean lifespan, then we should observe whether this added life accrues to H-span (healthy years) or to G-span (decrepit years).
Premise 3 (empirical finding): If most longevity interventions increase both mean lifespan and the fraction of life spent in decrepitude (G-span_rel), then lifespan extension is not purely "healthy" but often involves expanded late-life frailty.
Premise 4 (parameter association): If β consistently decreases when G-span_rel increases, then β reductions largely reflect heterogeneity in decrepitude (extended twilight longevity, ETL), not uniformly slower ageing.
Premise 5 (healthspan correlation): If α consistently decreases when absolute H-span increases, then α more directly indexes the timing of decrepitude onset and thus the "pace" of biological ageing.
Premise 6 (subpopulation effects): If β is always lower for the pooled cohort than for pathology-defined subpopulations, then β is sensitive to hidden heterogeneity in survival distributions (frailty mixture effect).
Then: (1) β should be reinterpreted as a proxy for demographic heterogeneity in late-life decrepitude (ETL) rather than the biological ageing rate; (2) α more closely reflects the pace of ageing, since it tracks expansion of healthy lifespan; and (3) therefore, the traditional reading of Gompertz parameters is inverted: α ≈ biological ageing pace, β ≈ survival heterogeneity.

Reviewer #2

(Remarks to the Author)
Zhang and Gems investigate, in C. elegans, the relationship between lifespan distributions and the relative proportions of healthspan and gerospan, in their manuscript entitled "Slow Gompertzian ageing in long-lived C. elegans results from expansion of decrepitude, not decelerated ageing."
By measuring lifespan, healthspan, gerospan, and tissue-specific infections under interventions including temperature shifts, antibiotics, and daf-2 mutations, they report several noteworthy findings:
1. Relative gerospan is positively correlated with lifespan and negatively correlated with the Gompertz timescale.
2. The previously described "extended twilight" phenomenon—where longer-lived individuals tend to have disproportionately longer gerospans—was amplified by interventions associated with reduced Gompertz aging rate (β, inverse of Gompertz timescale).
3. Healthspan correlates more strongly with the Gompertz shape parameter (α) than with the scale parameter (1/β).
4. Lifespan distribution shifts correspond with changes in both prevalence and age of tissue-specific infections.
From these observations, the authors question the traditional interpretation of Gompertz β as the sole demographic proxy of ageing rate and instead propose that α may be a more informative parameter.
While I remain unconvinced by some of the conclusions, the manuscript brings valuable data to an important question that is worth examining carefully. Understanding whether demographic mortality patterns truly reflect underlying biological ageing, or instead the dynamics of specific morbidities, is central to biodemography. The authors provide exactly the kind of individual-level measurements—lifespan, healthspan, and morbidity—that are needed to move the field forward.
Major comments
1. Parametric fitting and goodness-of-fit.
The reliance on Gompertz parametric fits throughout the analysis makes it essential that goodness-of-fit (GoF) be demonstrated rigorously. The Extended Data Fig. 2 suggests fits are strong when evaluated by $R^2$, but visual inspection alone and $R^2$ are not fully appropriate for survival distributions, which are constrained. Tests such as Kolmogorov–Smirnov or Anderson–Darling would provide a more robust evaluation. This point is important not as a technical detail, but because deviations from Gompertz have been repeatedly reported in many species, including C. elegans. Stronger statistical validation of fit would bolster confidence in the parametric comparisons presented.
2. Interpretation of β.
The manuscript makes a bold claim that reductions in Gompertz beta arise from expanded decrepitude rather than slowed ageing. The data clearly show a correlation between relative gerospan and β, but the conclusion that decrepitude expansion causes lower beta may be overstated.
3. Overall coherence.
The breadth of data is impressive, but the narrative sometimes risks overwhelming the reader. Much of the results section walks carefully through cohort-specific outcomes, yet the integrative message becomes harder to follow. A clearer synthesis —perhaps through schematic models or summary figures—could help the reader see how the many detailed findings converge on the authors' central proposal.
Line-by-line comments
- Line 80-89: There are quite significant methodological issues with the survival analysis described above. In addition, the Weibull model the authors cited in line 87-88 is not more complex than Gompertz (both have 2 parameters) but at least for some C. elegans survival curves generate better GoF. While I sympathize with the author's goals of finding biological basis for simple mortality models, the GoF presented here and in Extended Data Fig. 2 are not convincing.
- Line 151-168, Fig. 1f-h: The authors here is trying to discuss the trends of gerospan and correlations with aging rate

(parameter beta) in the Gompertz model, specifically arguing against a model of overall slowed pace of aging. While I can sympathize the idea that the data is much more complex than simple temporal scaling, the method and data interpretation is flawed: beta is not an independent variable, given that alpha is not held constant. If we look at the data itself (Extended Data Fig. 2) without the Gompertz model, for example at the first row (N2), it is hard to argue that lower temperature increased or held constant the rate of aging (stated on line 152 or shown in the 1st row of Fig. 1f).
- Line 173-176: The authors define inter-individual variability as variance/standard deviation in lifespan, whereas in reference 29 in which similar ideas are discussed, lifespan variability is measured in dimensionless, for example in CV.
- Line 178-180: I am confused by the logic here.
- Line 241-243, Line 251-253: The causal here is unwarranted. When concluding that beta reflect the inter interindividual variation, the authors are ignoring beta's effect on mean lifespan.
- Figure 2a: Negative correlation between beta and standard deviation follows from the definition of Gompertz law, I am not fully understanding what this panel is supposed to tell us.
- Figure 2: Many panels showing data in a nonstandard way, and only shows trends with no raw data.
- Line 300-305: this should be shown in a figure. The corresponding Figure 4 shows a lot of data but leave with the reader no tool to interpret that data.
- Line 316-322, Line 352-357: again, I don't agree with isolating of one demographic parameter, and interpret its correlation with health as causal relations.
- Line 540-542: raw data (n=8830) is not provided, only summary statistics. Critically, H-spans & G-spans of individual worms should be provided in some form to be consistent with the data availability statement here.

Reviewer #3

(Remarks to the Author)
Summary: In the provided paper, Zhang and Gemes investigate the demographic parameters alpha and beta of the Gompertz equation to identify their biological correlatives and to provide new insights into their interpretation. To quantify aging independent of the Gompertz parameters, they individually tracked the locomotory activity of each worm and scored their health based on the locomotion pattern. These quantifications enable them to determine the absolute and relative health and gerospan of the animals. They then measure health and gerospan across different lifespan-extending interventions, including three daf-2 mutations, wild-type animals, carbonite ceiling, different temperatures, and combinations thereof. Their data in Figures 1–3 provide the following conclusions: 1) Low beta values, traditionally associated with a lower aging rate, prolong the gerospan. 2) Beta is associated with a wider inter-individual variability of the gerospan. 3) Conversely, a low alpha is a (somewhat) better parameter to quantify absolute health span. In figures four and five, they then investigate how beta as a value relates to the prevalence of pathological subpopulations and analyze the different modes of death and how beta relates to their distribution across different lifespan extensions.

In general, the paper is well done. The studies are sufficiently powered, and the work conducted is extensive. This is a high-quality and informative study that sheds light on one of the most widely used mathematical models of aging. It empirically quantifies the relation of alpha and beta to the rate of aging.
Major criticisms: There is a lot to like in this study, and it is a significant step forward in biologically interpreting the parameters of the Gompertz equation. However, the authors do not explain how the original interpretation of beta as the rate of aging came about and why their updated explanation differs so much. The reason why the beta parameter in the Gompertz equation was equated with the rate of aging was that aging has been traditionally defined as the rate of all-cause mortality. Gompertz developed his equation to model mortality, with the original interpretation of beta as the rate of mortality with age. Based on the definition that aging is the rate of mortality, beta remains the rate of aging. However, what this older interpretation of aging implied—a belief that is unequivocally refuted by the current work—was that a lower rate of aging is associated with greater health. In this manuscript, the authors start out by redefining aging through the measure of locomotion. They then quantify health span and gero-span based on this new measure that is no longer based on all-cause mortality but on a measure of organismal health. It is the redefinition of aging based on health parameters rather than mortality that consequently disrupts the association of beta with aging. Redefining aging by including various health parameters is a perfectly reasonable approach and one that has become increasingly popular in the aging field, and a view that probably supplanted all-cause mortality as the key defining feature of aging. However, the authors should clearly state that the definition of beta as a measure of the rate of aging is dependent on one's definition of aging. By the definition of all-cause mortality, beta remains a measure of the rate of aging. Conversely, defining aging based on declining health—a more current interpretation of how the field views aging—changes the interpretation of beta, and it becomes a measure of inter-organismal variability and a measure of the time spent in decrepitude. It is crucial to make clear to readers who are less familiar with the intricacies of the Gompertz equation that the precise interpretation of beta inherently depends on the definition of aging.

Minor criticisms:
1) In Figure 2, the unit for absolute lifespan is missing and should be indicated, which is presumably days. See y-axis Figure 2,b c, d, e, f & g, Y-axis: absolute health-span and gero-span. The unit is missing, which is probably days.
2) In Figure 4C, panel daf-2(e1370) big P @ 25°C. The lifespan curve seems to be missing. Is that because there are no big P deaths at 25°, or is that an omission? Please clarify.

Version 1:

Reviewer comments:

Reviewer #1

(Remarks to the Author)
I accept the author's defence of temperature as a mechanistic modifier by showing it disproportionately expands relative gerospan in 12 of 16 treatments and alters specific pathological trajectories. Citing Xiao et al. (2013) and Vakkayil and Hoppe (2022) establishes that temperature regulates longevity through specific genetic and proteostatic programs. The authors also clearly distinguish their work from traditional (Vauple) frailty theory. While Vaupel suggests beta reflects the aging rate within hidden subpopulations, their study demonstrates that beta primarily measures inter-individual heterogeneity in late-life decrepitude (gerospan). Thus, beta is a metric of survival dispersion rather than biological aging pace.

I also accept their arguments regarding reducing some of the complexity in the manuscript write up. But it will be a heavy lift for some readers.

Reviewer #2

(Remarks to the Author)
Upon reading the revised manuscript and the authors' responses, I find that the manuscript has been substantially improved. In the original version, my concerns were largely technical and stemmed from difficulty in following the central conceptual picture and assessing the statistical adequacy of the numerous sub-claims within a complex set of experiments and data. The revised manuscript now presents the experiments and results in a much clearer and more coherent manner, and several of my methodological concerns have been addressed directly through additional analyses, sharing of original data and improved presentation.
In particular, the authors' central proposal—that reductions in the Gompertz β parameter reflect heterogeneous expansion of gerospan more than extension of healthspan—is now articulated more transparently. The connection between the empirical findings and the reinterpretation of demographic aging parameters is clearer and better supported by the data.

Major points from previous review
1. Parametric fitting and goodness-of-fit
I requested more rigorous goodness-of-fit evaluation for the Gompertz modeling. The authors have now implemented Kolmogorov–Smirnov and Anderson–Darling tests and reported these results. While deviations remain in some cohorts (as expected), the additional statistical treatment is appropriate and sufficient for the purposes of the manuscript, which focuses on the biological interpretation of Gompertz parameters rather than on defending Gompertz as a universally exact statistical model.
2. Interpretation of β
I previously raised concerns that the manuscript risked overstating causality in linking reduced β to expanded decrepitude. The revised text now clarifies the level of causation being invoked (life-course/mathematical rather than mechanistic), and this distinction is adequately explained. The claim is more carefully framed and appropriately supported.
3. Narrative coherence
The manuscript is now conceptually much clearer. The addition of a summarizing schematic (Fig. 5e) and restructuring of the Results section substantially improve readability and transparency of the authors' argument. The central message—that demographic slowing (reduced β) in these experiments corresponds primarily to gerospan expansion rather than healthspan extension—is now presented in a coherent and integrated manner.
Remaining points
I still have the following minor reservations regarding aspects of presentation, but these are not central to the overall conclusions. At this stage, I do not believe further line-by-line revision is necessary.
1. Scope of individual-level analyses (Fig. 2b–g; Results section iii).
Individual-level gerospan and healthspan analyses are presented for a subset of 21 interventions (8/16 reduced temperature, 6/12 carbenicillin treatments, 7/18 other interventions) out of the 46 total cohorts analyzed in the study. Moreover, the extended twilight longevity (ETL) pattern is evident in 15 of these 21. Thus, the claim that reduced β primarily reflects gerospan expansion is directly demonstrated for this subset rather than across all interventions. It may improve transparency to qualify this point more explicitly in the text.
2. Figure organization and panel structure.
I appreciate the substantial effort to reorganize the figures, and the manuscript is now much easier to follow. However, the ordering and placement of some sub-panels remain nonlinear and could still pose challenges for first-time readers. Further minor streamlining or clearer visual grouping of related panels might enhance accessibility.
I believe the manuscript is now suitable for publication, pending minor editorial adjustments as deemed appropriate.

Reviewer #3

(Remarks to the Author)
The authors have thoroughly addressed my concerns, and I fully endorse publication of this paper. Their revisions were

thoughtful, comprehensive, and clearly strengthened the overall quality and clarity of the work.

**Open Access** This Peer Review File is licensed under a Creative Commons Attribution 4.0 International License, which permits use, sharing, adaptation, distribution and reproduction in any medium or format, as long as you give appropriate credit to the original author(s) and the source, provide a link to the Creative Commons license, and indicate if changes were made.
In cases where reviewers are anonymous, credit should be given to 'Anonymous Referee' and the source.
The images or other third party material in this Peer Review File are included in the article's Creative Commons license, unless indicated otherwise in a credit line to the material. If material is not included in the article's Creative Commons license and your intended use is not permitted by statutory regulation or exceeds the permitted use, you will need to obtain permission directly from the copyright holder.
To view a copy of this license, visit https://creativecommons.org/licenses/by/4.0/

**Author responses to reviewer comments**

NCOMMS-25-70240 “Slowed Gompertzian ageing in long-lived *C. elegans* results from expansion of decrepitude, not decelerated ageing”

**Reviewer #1:**

This study reinterprets Gompertz mortality parameters in C. elegans. Contrary to convention, it argues that reductions in β reflect heterogeneity and extended late-life decrepitude (gerospan), while reductions in α better track healthspan extension and slower biological ageing. Linking longitudinal locomotor health to survival, the authors propose α, not β, captures ageing pace.

There is little doubt that this paper merits publication. In fact, it reads almost as two papers in one: a hierarchy in which one layer generates evidence for reinterpretation of the Gompertz parameters, and another delves deeply into the biology of ageing. The breadth of the experiments and the strength of the data are remarkable. Yet unless a study of this complexity is presented with exceptional clarity, its impact beyond the inner sanctum of cell and molecular biology, reaching, for example, demographers and population biologists, will remain limited. Demographers in particular, are unlikely to fully understand or even engage with the results without clear guidance through the visual and conceptual density.

Author response: We are thankful for the reviewer’s acknowledgment of the work’s data quality and interdisciplinary impact, and have endeavoured to improve its accessibility for biologists and demographers alike, as detailed below.

In addition to the need to make the paper more accessible (outlined later), I have two conceptual concerns that I would like the authors to address more clearly in the manuscript.

1. Justification for temperature as treatment. While reduced temperature reliably extends C. elegans lifespan, in my view its interpretive value for ageing is limited. Lower temperature slows virtually all biological processes including development, reproduction, and decline through thermodynamic effects, akin to redefining a “day” as 12 hours instead of 24. The clock runs more slowly, so individuals live longer in calendar time, but nothing has fundamentally changed about the mechanisms of ageing. By contrast, interventions such as dietary restriction or daf-2 mutations alter how long worms remain healthy relative to their developmental and reproductive schedules. These pathway-level manipulations reflect genuine changes in ageing pace, whereas temperature effects reflect environmental pacing. Thus, cold exposure demonstrates lifespan plasticity but is a weaker test of mechanistic theories of ageing. The authors cite no papers on temperature as a life-extending method that situates it as an slowed aging concept.

Author response: We agree with the reviewer that reduced temperature may extend *C. elegans* lifespan largely through thermodynamic effects on the pace of life, and that this could alter the speed rather than mechanisms of ageing. However, we consider both (changes in speed or mechanism) to be true changes in the ageing process. In fact, changes in the speed (rate) of ageing are exactly what the $\beta$ parameter has traditionally been thought to capture. Thus, we believe that temperature is a fitting treatment in this study, as it is a putative modulator of the rate of ageing.

In addition, our results in fact indicate that temperature controls not only the rate but mechanisms of ageing (which we did not expect). For instance, in 12/16 lower temperature treatments, relative G-span was increased, while lowering temperature changed the prevalence of end-of-life pathologies (decreasing and increasing pharyngeal and intestinal bacterial colonisation, respectively). This argues that temperature has modified the mechanistic trajectory of ageing. We have now also cited two other studies that view temperature as a modulator of *C. elegans* ageing beyond thermodynamic effects, at first introduction of temperature as a treatment of interest:

1. Xiao et al. (2013) "A genetic program promotes *C. elegans* longevity at cold temperatures via a thermosensitive TRP channel". *Cell*, 152:806–817.
2. Vakkayil and Hoppe (2022) "Temperature-dependent regulation of proteostasis and longevity". *Frontiers in Aging*, 3:853588.

2. Frailty versus gerospan. The authors reinterpret β as a marker of heterogeneity in gerospan rather than as the rate of biological aging. Although they cite Vaupel's 1979 frailty model, they do not engage with its central implication that β still represents the rate of aging within sub-cohorts, with the pooled β emerging from their mixture. By contrast, their gerospan framework discards this interpretation altogether, treating β solely as a metric of variation in decrepitude across individuals. Thus, both models place heterogeneity at the center of demographic aging, but they diverge sharply on whether β should be viewed as a rate of aging or a measure of dispersion. The authors need to make this distinction clear so readers can understand how their reinterpretation departs from established demographic theory.

Author response: This is indeed a key distinction between our new view of $\beta$ and existing views of $\beta$ from frailty/heterogeneity models. We are glad the reviewer has noted this subtle but critical detail and alerted us to its need for greater clarity. We have now stated this explicitly in the discussion, as follows:

"Notably, our reinterpretation of $\beta$ differs from that of existing heterogeneity-based models, e.g. Vaupel et al. (1979), which assume that at least within subpopulations, $\beta$ does reflect biological ageing rate. In contrast, our data suggest that $\beta$ better reflects heterogeneity than biological ageing rate between and within subpopulations."

I see two levels of clarity that need to be improved in the manuscript. The first concerns the overarching hypothesis or framework. With 46 treatments and a bewildering number of

"moving parts" (graphs; sub-graphs; sub-sub-graphs), the big experimental picture is extremely difficult to grasp. Readers would benefit from a clear, anchoring framework, something akin to a guiding syllogism ("if premises n are met, then X, Y, Z"), that makes the logical structure of the argument explicit. Such a framework would help orient the reader as they work their way through the results.

Author response: We appreciate that the multidimensionality of our experimental design and analyses is challenging for readers. We understand that this challenge lies not with grasping the overarching aim of the study (to determine the biological meaning of the Gompertz parameters), but rather how each experiment/analysis contributes towards the overarching aim. We therefore thank the reviewer for their detailed and precise suggestion of possible guiding syllogisms (see final reviewer 1 paragraph below). We have now incorporated similar guiding statements where relevant when introducing the results sections. We have also added the following primer at the end of the introduction, informing readers of the component aims, how they are logically connected, how the results will be structured accordingly, and refer them to the summary figure:

"Specifically, we investigated: (1) how life-extension affects the duration of healthy versus decrepit life, (2) whether such changes in health/decrepitude correlate with changes in the Gompertz parameters, (3) whether these correlations can be explained causally, and (4) whether the Gompertz parameters can be causally related to specific age-related pathologies. We will present our results (summarised in Fig. 5e) in the order of these aims."

The second concerns the details (i.e., Results). The figures in this study demand readers process patterns at several levels: within plots (curvature of survival curves, divergence of lines), between subfigures (temperature vs. antibiotic vs. daf-2 effects), and across figures (gerospan expansion, variability, α vs. β, necropsy subpopulations). They must parse directional shifts (α vs. β), proportional changes in healthspan vs. gerospan, cross-condition contrasts, subpopulation divergences, statistical signals, and conceptual overlays like "extended twilight longevity." The cumulative expectation is that readers track 46 treatments, distinguish absolute from relative measures, reinterpret α and β, connect demographic curves with individual outcomes, and integrate diverse statistical modes—spread across 5 figures with ~58 distinct plots. In sum, this is a bewildering interpretive task requiring alignment of scales, metrics, and interventions. For this reason, I strongly recommend that the authors revisit the Results and consider a clearer, more structured way of presenting both the figures and the accompanying narrative, so that readers outside of the immediate field can engage with and appreciate the significance of their findings. I understand this will be no easy task, but without a clearer and more structured presentation, many readers will be left unable to follow the argument and may simply defer to the authors' authority rather than genuinely engaging with the evidence.

Author response: The reviewer accurately describes the complexity of our dataset and results, which stem primarily from their multidimensionality and inter-relatedness. In presenting our

results, we have aimed to strike a careful balance between simplicity and transparency. For instance, we have considered replacing the various multi-panel figures for the 3 treatment classes (low temperature, antibiotic, *daf-2* mutation) with single figures portraying the overall pattern (e.g. most common effect of life-extension on relative gerospan, rather than displaying the pattern for each individual treatment), and/or moving the more complex multi-panel figures to the supplement. However, we realised that this could discourage readers from actively engaging with the data, particularly if its full details were in the supplement. This study has been possible through careful re-evaluation and re-definition of existing ideas, which required an understanding of the boundaries of processes and concepts, and importantly, their exceptions. We therefore feel that the precision preserved within our multi-panel figures is necessary for the interested reader to engage scientifically with the data.

We have therefore employed a hybrid strategy with several ways of improving readability of the Results:

(1) The now improved accompanying narrative (see previous author response) we believe significantly improves structure and clarity.

(2) The accompanying narrative now always summarises the overall patterns from the multi-panel figures (e.g. "44/46 treatments increased mean lifespan").

(3) The multi-panel figures always appear in the same general format.

(4) We have removed repetitive/redundant y-axis labels in Fig. 1b–h and 2c–g to reduce visual clutter, and colour-coded the two y-axis labels ($\beta$ and Relative G-span) in Fig. 1f–h for reader convenience.

(5) "ETL" (extended twilight longevity) vs "non-ETL" treatments have been annotated in Fig. 2b–g, to help readers connect between the main text and figure.

(6) The term "inverted ETL" has been removed from Results section (iii) to reduce the number of new terms, and its explanation rewritten to be simpler (yet still precise).

Possible syllogism:
Premise 1 (health vs. decrepitude): If locomotor health can be separated into two phases, H-span and G-span, then we can track healthspan and decrepitude separately across lifespan interventions.
Premise 2 (effects of interventions): If interventions such as lower temperature, antibiotics, and daf-2 mutations extend mean lifespan, then we should observe whether this added life accrues to H-span (healthy years) or to G-span (decrepit years).
Premise 3 (empirical finding): If most longevity interventions increase both mean lifespan and the fraction of life spent in decrepitude (G-span_rel), then lifespan extension is not purely "healthy" but often involves expanded late-life frailty.
Premise 4 (parameter association): If β consistently decreases when G-span_rel increases, then β reductions largely reflect heterogeneity in decrepitude (extended twilight longevity, ETL), not uniformly slower ageing.
Premise 5 (healthspan correlation): If α consistently decreases when absolute H-span increases, then α more directly indexes the timing of decrepitude onset and thus the "pace" of biological ageing.
Premise 6 (subpopulation effects): If β is always lower for the pooled cohort than for

pathology-defined subpopulations, then β is sensitive to hidden heterogeneity in survival distributions (frailty mixture effect).
Then: (1) β should be reinterpreted as a proxy for demographic heterogeneity in late-life decrepitude (ETL) rather than the biological ageing rate; (2) α more closely reflects the pace of ageing, since it tracks expansion of healthy lifespan; and (3) therefore, the traditional reading of Gompertz parameters is inverted: α ≈ biological ageing pace, β ≈ survival heterogeneity.

Author response: We again thank the reviewer for this very helpful sequence of possible syllogisms. Please see our author response at the start of page 3.

**Reviewer #2:**

Zhang and Gems investigate, in C. elegans, the relationship between lifespan distributions and the relative proportions of healthspan and gerospan, in their manuscript entitled "Slow Gompertzian ageing in long-lived C. elegans results from expansion of decrepitude, not decelerated ageing."
By measuring lifespan, healthspan, gerospan, and tissue-specific infections under interventions including temperature shifts, antibiotics, and daf-2 mutations, they report several noteworthy findings:
1. Relative gerospan is positively correlated with lifespan and negatively correlated with the Gompertz timescale.
2. The previously described "extended twilight" phenomenon—where longer-lived individuals tend to have disproportionately longer gerospans—was amplified by interventions associated with reduced Gompertz aging rate (β, inverse of Gompertz timescale).
3. Healthspan correlates more strongly with the Gompertz shape parameter (α) than with the scale parameter (1/β).
4. Lifespan distribution shifts correspond with changes in both prevalence and age of tissue-specific infections.
From these observations, the authors question the traditional interpretation of Gompertz β as the sole demographic proxy of ageing rate and instead propose that α may be a more informative parameter.
While I remain unconvinced by some of the conclusions, the manuscript brings valuable data to an important question that is worth examining carefully. Understanding whether demographic mortality patterns truly reflect underlying biological ageing, or instead the dynamics of specific morbidities, is central to biodemography. The authors provide exactly the kind of individual-level measurements—lifespan, healthspan, and morbidity—that are needed to move the field forward.

Author response: We thank the reviewer for their constructive feedback, and are grateful for their appreciation of the work's relevance for advancing the field.

Major comments

1. Parametric fitting and goodness-of-fit.
The reliance on Gompertz parametric fits throughout the analysis makes it essential that goodness-of-fit (GoF) be demonstrated rigorously. The Extended Data Fig. 2 suggests fits are strong when evaluated by $R^2$, but visual inspection alone and $R^2$ are not fully appropriate for survival distributions, which are constrained. Tests such as Kolmogorov–Smirnov or Anderson–Darling would provide a more robust evaluation. This point is important not as a technical detail, but because deviations from Gompertz have been repeatedly reported in many species, including C. elegans. Stronger statistical validation of fit would bolster confidence in the parametric comparisons presented.

Author response: This is a very helpful methodological suggestion. We have now run both Kolmogorov–Smirnov and Anderson–Darling goodness-of-fit tests. The latter assesses global fit across all ages and finds almost all cohorts to be well-fit by Gompertz. We note that this test is also more sensitive to tail deviations, which is where deviations from Gompertz often occur. The former test (Kolmogorov-Smirnov) identified deviations from Gompertz in a number of cohorts (approximately half); however this is not unexpected given that this test focuses on the region of worst fit, rather than global fit. We present these statistics in Supplementary Table 2 and as follows in the main text (just after the $R^2$ results):

"Anderson-Darling goodness-of-fit tests also supported these Gompertzian fits in most cohorts (21–22/24) despite localised deviations within some (Supplementary Table 2)."

2. Interpretation of β.
The manuscript makes a bold claim that reductions in Gompertz beta arise from expanded decrepitude rather than slowed ageing. The data clearly show a correlation between relative gerospan and β, but the conclusion that decrepitude expansion causes lower beta may be overstated.

Author response: We appreciate that the distinction between correlation and causation must be navigated carefully here. In Results section (ii) (Fig. 1f–g) we present the correlation between increased gerospan and decreased $\beta$, and in section (iii) (Fig. 2) we explain how this correlation arises (i.e. can we demonstrate causality?). We show in several ways that lifespan extension in longer-lived population members (in/closer to the survival curve tail) results primarily from gerospan extension (rather than healthspan extension). This means that the 'stretching out' (i.e. $\beta$ reduction) of these survival curves is in fact caused by the gerospan expansion event. Here, causation is understood in a life-course/mathematical way (changes in lifespan are caused by additive changes in healthspan and/or gerospan). We summarise this idea visually in Fig. 5e.

But we do understand that there can be other levels of causality. For instance, there are also more biologically-mechanistic causes of $\beta$ reduction, such as the

molecular/cellular/systemic processes that directly determine lifespan and therefore $\beta$. However, as we understand it, this is a different hypothesis and one that we have been careful not to claim as our focus.

3. Overall coherence.
The breadth of data is impressive, but the narrative sometimes risks overwhelming the reader. Much of the results section walks carefully through cohort-specific outcomes, yet the integrative message becomes harder to follow. A clearer synthesis—perhaps through schematic models or summary figures—could help the reader see how the many detailed findings converge on the authors' central proposal.

Author response: We have now improved the readability and clarity of our central findings. In particular, Fig 5e provides a clear summary schematic of our central findings, which we refer readers to at the end of the introduction.

We list here other improvements to this end:
(1) A list of key aims (same order as results section) is presented at the end of the introduction, and the results section openings have been adjusted to match this.
(2) The accompanying narrative now always summarises the overall patterns from the multi-panel figures (e.g. "44/46 treatments increased mean lifespan").
(3) The multi-panel figures always appear in the same general format.
(4) We have removed repetitive/redundant y-axis labels in Fig. 1b–h and 2c–g to reduce visual clutter, and colour-coded the two y-axis labels ($\beta$ and Relative G-span) in Fig. 1f–h for reader convenience.
(5) "ETL" (extended twilight longevity) vs "non-ETL" treatments have been annotated in Fig. 2b–g, to help readers connect between the main text and figure.
(6) The term "inverted ETL" has been removed from Results section iii to reduce the number of new terms, and its explanation rewritten to be simpler (yet still precise).
(7) More detailed cohort-specific descriptions have been streamlined to focus attention on the integrative message.

Line-by-line comments
- Line 80-89: There are quite significant methodological issues with the survival analysis described above. In addition, the Weibull model the authors cited in line 87-88 is not more complex than Gompertz (both have 2 parameters) but at least for some C. elegans survival curves generate better GoF. While I sympathize with the author's goals of finding biological basis for simple mortality models, the GoF presented here and in Extended Data Fig. 2 are not convincing.

Author response: We thank the reviewer for this constructive feedback. We have now addressed this concern with additional goodness-of-fit tests (Kolmogorov-Smirnov and Anderson-Darling tests); please see our earlier response on page 6.

Regarding Vanfleteren et al. (1998), we have cited this in reference to the three-parameter logistic model that was used (in addition to two-parameter logistic and Weibull models). However, to improve precision, we have amended our sentence as follows:

"Although other mortality models can better fit…", instead of "Although more complex mortality models can better fit…"

- Line 151-168, Fig. 1f-h: The authors here is trying to discuss the trends of gerospan and correlations with aging rate (parameter beta) in the Gompertz model, specifically arguing against a model of overall slowed pace of aging. While I can sympathize the idea that the data is much more complex than simple temporal scaling, the method and data interpretation is flawed: beta is not an independent variable, given that alpha is not held constant. If we look at the data itself (Extended Data Fig. 2) without the Gompertz model, for example at the first row (N2), it is hard to argue that lower temperature increased or held constant the rate of aging (stated on line 152 or shown in the 1st row of Fig. 1f).

Author response: This is a complex concern to which we will respond with care. We apologise for the long response.

In the Results section (ii) we presented how each life-extending treatment affects $\beta$ and relative gerospan (e.g. Fig. 1f–h) to ask a simple question: when $\beta$ changes, what happens to relative gerospan? We are interested in the latter because it is one readout of the biological ageing process: assuming a simple slowing of the overall ageing process, relative gerospan (proportion of life spent in gerospan) should not change, since all life stages would be stretched in proportion. Therefore, our finding that life-extending treatments simultaneously decrease $\beta$ and *increase* relative gerospan suggests that these $\beta$ reductions do not reflect a simple slowing of biological ageing rate.

Here there are two separate claims about biological ageing rate. The observed relative gerospan expansion suggests that (1) ageing rate is not simply slowed as though stretching an elastic string, and (2) $\beta$ does not reflect such a mode of slowed ageing (which is what it is often thought to reflect). Here we are essentially playing Devil's advocate, to test our hypothesis that the traditional interpretation of $\beta$ is too simple to explain real data. Our finding of gerospan expansion suggests a more complex form of ameliorated ageing, that increases lifespan more than healthspan.

We appreciate that a single demographic change in lifespan (and therefore the ageing process) shapes both $\alpha$ and $\beta$, such that it can be difficult or seemingly fallacious to determine the relative contribution to each parameter. However, assuming correct model fitting, by breaking down the ageing process into biologically-distinct components (i.e. healthspan vs. gerospan, or infected vs non-infected individuals), it becomes possible to start exploring how these components might interact (in an additive-like manner) to produce the observed Gompertz parameters. Indeed, we find relationships between $\alpha$ and mean healthspan, and between $\beta$ and gerospan variation, and these relationships do not depend on each other (e.g. if a treatment changed only one parameter, the relationship would still be true for this parameter). We depict this idea in summary Fig. 5e, showing a treatment that decreases both

$\alpha$ and $\beta$, reflecting respectively, inter-individually invariant healthspan expansion combined with inter-individually variable gerospan expansion.

Additionally, traditional biological interpretation of the Gompertz parameters ($\alpha$: ageing-independent life-limiting processes and $\beta$: ageing rate-determining processes) assume independency of their mechanisms, and these are indeed interpreted independently (e.g. there are simultaneously increased ageing-independent processes and decreased ageing-rate processes). In order to test these views and re-interpret the Gompertz parameters, we follow the template of $\alpha$=X, $\beta$=Y. We are, however, aware that the Gompertz parameters are just a tool, and the biological processes (X, Y) can readily be reassigned to the parameters of any other well-fitting mortality model.

Regarding Line 152, it states that $\beta$ (not ageing rate) is increased or held constant by lowered temperature (first column rather than row of Fig. 1f).

- Line 173-176: The authors define inter-individual variability as variance/standard deviation in lifespan, whereas in reference 29 in which similar ideas are discussed, lifespan variability is measured in dimensionless, for example in CV.

Author response: That is correct, we have quantified absolute rather than relative (dimensionless) variation. The two measure slightly different things: absolute variation (e.g. standard deviation) directly measures variation, whereas relative variation (e.g. coefficient of variation) measures the variation relative to the mean. The latter assumes biological relevance of some interaction between variation and mean. In our study, we preferred the biologically simpler and intuitive measure of absolute variation, which importantly enabled us to relate the Gompertz parameters to inter-individual variation in healthspan/gerospan in a tractable way.

- Line 178-180: I am confused by the logic here.

Author response: We have rewritten this section to be clearer:

"An often-overlooked mathematical property of the Gompertz rate parameter $\beta$ is its inverse relation to lifespan variation, which in comparison is only marginally affected by the $\alpha$ parameter[42] (Fig. 1a, fourth panel). Indeed, amongst our 24 treatments, $\beta$ showed a strong inverse correlation with lifespan standard deviation (Fig. 2a).

Given this, we wondered whether this increased lifespan variation in small-$\beta$ cohorts could result from increased inter-individual variation in the ageing process. Interestingly, it was previously estimated that G-span differences account for most (~67%) inter-individual lifespan variation in *C. elegans*[21]. Thus, the correlation between reduced $\beta$ and increased G-span$^{rel}$ in our cohorts may arise from inter-individually variable G-span expansion."

- Line 241-243, Line 251-253: The causal here is unwarranted. When concluding that beta reflect the inter interindividual variation, the authors are ignoring beta's effect on mean lifespan.

Author response: Please see our earlier response to the comment on correlation vs. causation, which we address on page 6.

In our model of $\beta$ reflecting inter-individual gerospan variation, we do account for $\beta$'s effect on mean lifespan, because our model proposes that inter-individually variable expansion of gerospan decreases $\beta$. Here, mean lifespan is increased due to the inter-individually variable gerospan expansion event. This idea is summarised visually in Fig. 5e.

- Figure 2a: Negative correlation between beta and standard deviation follows from the definition of Gompertz law, I am not fully understanding what this panel is supposed to tell us.

Author response: We include this figure to illustrate this mathematical relationship between $\beta$ and lifespan variation for those readers from less mathematical/demographic backgrounds. The relationship between $\beta$ and lifespan variation may not be immediately obvious to all within the diverse field of ageing research. It also helps establish our motivation to focus on inter-individual variation, in not only lifespan but the preceding ageing process.

Additionally, this plot provides a reassuring sanity check for the reader that the data and model are behaving as expected, before delving into the more complex analyses.

- Figure 2: Many panels showing data in a nonstandard way, and only shows trends with no raw data.

Author response: We have presented these data in non-standard ways due to the unconventional questions that we have asked. We chose modes of data presentation that most easily communicate our findings to the reader. The multidimensional dataset (all combinations of temperature, antibiotic status and genotype) also poses a challenge for data presentation, which accounts for the large number of related panels. To help readers connect between the main text and figure, "ETL" (extended twilight longevity) vs "non-ETL" treatments have been annotated in panels b–g.

We provide a table of raw data sufficient to reproduce these figures, which includes healthspan and gerospan values for each individual where scored. We have remembered to attach this file in our resubmission.

- Line 300-305: this should be shown in a figure. The corresponding Figure 4 shows a lot of data but leave with the reader no tool to interpret that data.

Author response: This paragraph describes the $\beta$ values of the whole populations and their component subpopulations. We feel that within the formatting constraints, these $\beta$ values are most effectively communicated in table format (Supplementary Table 7).

To our knowledge, this paragraph does not directly correspond to Figure 4.

- Line 316-322, Line 352-357: again, I don't agree with isolating of one demographic parameter, and interpret its correlation with health as causal relations.

Author response: Please see our earlier responses about independent interpretation of parameters (page 8) and correlation vs. causality (page 6).

- Line 540-542: raw data (n=8830) is not provided, only summary statistics. Critically, H-spans & G-spans of individual worms should be provided in some form to be consistent with the data availability statement here.

Author response: We thank the reviewer for drawing out attention to this. We have now remembered to attach the raw data, which can be viewed in the resubmission. It includes lifespan, healthspan, gerospan and necropsy corpse type for all individuals where those traits were measured.

**Reviewer #3:**

Summary: In the provided paper, Zhang and Gemes investigate the demographic parameters alpha and beta of the Gompertz equation to identify their biological correlatives and to provide new insights into their interpretation. To quantify aging independent of the Gompertz parameters, they individually tracked the locomotory activity of each worm and scored their health based on the locomotion pattern. These quantifications enable them to determine the absolute and relative health and gerospan of the animals. They then measure health and gerospan across different lifespan-extending interventions, including three daf-2 mutations, wild-type animals, carbonite ceiling, different temperatures, and combinations thereof. Their data in Figures 1–3 provide the following conclusions: 1) Low beta values, traditionally associated with a lower aging rate, prolong the gerospan. 2) Beta is associated with a wider inter-individual variability of the gerospan. 3) Conversely, a low alpha is a (somewhat) better parameter to quantify absolute health span. In figures four and five, they then investigate how beta as a value relates to the prevalence of pathological subpopulations and analyze the different modes of death and how beta relates to their distribution across different lifespan extensions.

In general, the paper is well done. The studies are sufficiently powered, and the work conducted is extensive. This is a high-quality and informative study that sheds light on one of the most widely used mathematical models of aging. It empirically quantifies the relation of alpha and beta to the rate of aging.

Author response: We are grateful for the reviewer's appreciation of the work's relevance to the field, and thank them for their insightful feedback below.

Major criticisms: There is a lot to like in this study, and it is a significant step forward in biologically interpreting the parameters of the Gompertz equation. However, the authors do not explain how the original interpretation of beta as the rate of aging came about and why their updated explanation differs so much. The reason why the beta parameter in the Gompertz equation was equated with the rate of aging was that aging has been traditionally defined as the rate of all-cause mortality. Gompertz developed his equation to model mortality, with the original interpretation of beta as the rate of mortality with age. Based on the definition that aging is the rate of mortality, beta remains the rate of aging. However, what this older interpretation of aging implied—a belief that is unequivocally refuted by the current work—was that a lower rate of aging is associated with greater health. In this manuscript, the authors start out by redefining aging through the measure of locomotion. They then quantify health span and gero-span based on this new measure that is no longer based on all-cause mortality but on a measure of organismal health. It is the redefinition of aging based on health parameters rather than mortality that consequently disrupts the association of beta with aging. Redefining aging by including various health parameters is a perfectly reasonable approach and one that has become increasingly popular in the aging field, and a view that probably supplanted all-cause mortality as the key defining feature of aging. However, the authors should clearly state that the definition of beta as a measure of the rate of aging is dependent on one's definition of aging. By the definition of all-cause mortality, beta remains a measure of the rate of aging. Conversely, defining aging based on declining health—a more current interpretation of how the field views aging—changes the interpretation of beta, and it becomes a measure of inter-organismal variability and a measure of the time spent in decrepitude. It is crucial to make clear to readers who are less familiar with the intricacies of the Gompertz equation that the precise interpretation of beta inherently depends on the definition of aging.

Author response: We thank the reviewer for this incisive and detailed feedback. This is indeed a core conceptual foundation of the work: the relationship and distinction between what we have called "demographic ageing" and "biological ageing", which we believe are the two definitions of ageing that the reviewer refers to (relating to the rate of all-cause mortality and health measures, respectively). We have made clear this distinction in the introduction as follows, informing readers of which definition (i.e. biological ageing) we are interested in:

"Though biological ageing affects individuals, researchers investigating it frequently use demographic (population) ageing as a metric[2,3]. However, demographic metrics (e.g. changes in mean lifespan) are difficult to link to determinative biological mechanisms.

This is true of a salient and enigmatic feature of demographic ageing: the exponential increase in all-cause mortality rate during adulthood, that is common (though not universal) among animal species[4,5]….

But to what do these demographic parameters correspond in terms of biological ageing, within individuals? … $\beta$ describes the rate of mortality rate increase, and because mortality rate increase has been traditionally defined as ageing, $\beta$ has thus become widely

interpreted as capturing the rate of ageing. Here, equivalence between demographic ageing (mortality rate increase) and biological ageing (rate of ageing) is assumed."

We have also ensured to specify "biological ageing" and "demographic ageing", rather than just "ageing", throughout the manuscript.

Minor criticisms:
1) In Figure 2, the unit for absolute lifespan is missing and should be indicated, which is presumably days. See y-axis Figure 2,b c, d, e, f & g, Y-axis: absolute health-span and gero-span. The unit is missing, which is probably days.

Author response: We have remade these y-axes to include the units (days).

2) In Figure 4C, panel daf-2(e1370) big P @ 25°C. The lifespan curve seems to be missing. Is that because there are no big P deaths at 25°, or is that an omission? Please clarify.

Author response: That is correct, there were no *daf-2(e1370)* P deaths at 25˚C. We have now added the following to the figure caption to make this immediately clear:
"At 25˚C, no *daf-2(e1370)* P deaths were observed, as previously noted(Zhao et al., 2021)."

**Author responses to Reviewer 2**

I still have the following minor reservations regarding aspects of presentation, but these are not central to the overall conclusions. At this stage, I do not believe further line-by-line revision is necessary.

1. Scope of individual-level analyses (Fig. 2b–g; Results section iii).
Individual-level gerospan and healthspan analyses are presented for a subset of 21 interventions (8/16 reduced temperature, 6/12 carbenicillin treatments, 7/18 other interventions) out of the 46 total cohorts analyzed in the study. Moreover, the extended twilight longevity (ETL) pattern is evident in 15 of these 21. Thus, the claim that reduced β primarily reflects gerospan expansion is directly demonstrated for this subset rather than across all interventions. It may improve transparency to qualify this point more explicitly in the text.

Author response: We thank the reviewer for this additional feedback. We specify that this pattern applies only to a majority subset of the 21 treatments, rather than all, as follows in the main text:

"Therefore, $\beta$ reduction reflected ETL in 5/8 reduced temperature, 6/6 antibiotic, and 4/7 *daf-2(rf)* treatments (15/21 or 71%, in total) where $\beta$ reduction co-occurred with G-span$^{rel}$ increase. In the 6 non-ETL exceptions (Fig. 2b, e–g), G-span$^{abs}$ still increased mostly in longer-lived population members (i.e. reducing $\beta$), in particular 15˚C treatment in *daf-2(e1368)* and *daf-2(e1370)* (Fig. 2b) and *daf-2(e1370)* treatment at 15˚C on carbenicillin (Fig. 2e). However, this was also largely true of H-span$^{abs}$; thus, in this minority of treatments, $\beta$ reduction reflects inter-individually variable expansion of both G-span and H-span."

2. Figure organization and panel structure.
I appreciate the substantial effort to reorganize the figures, and the manuscript is now much easier to follow. However, the ordering and placement of some sub-panels remain nonlinear and could still pose challenges for first-time readers. Further minor streamlining or clearer visual grouping of related panels might enhance accessibility.

Author response: We appreciate the reviewer's further suggestions regarding visual clarity and flow. We have worked to format the figures as intuitively as possible, while accommodating editorial formatting policies. In particular, we have used consistent and simple colour schemes to ensure clarity, a consistent layout of our multi-panel figures which readers will become familiar with throughout the study, and introduce figure panels in the same order that they appear in the text. However, we do understand that there will be an inevitable "reading curve" that accompanies this complex study, but for which we have done our best to lower.

I believe the manuscript is now suitable for publication, pending minor editorial adjustments as deemed appropriate.
